# Genomic Features of the Human Dopamine Transporter Gene and Its Potential Epigenetic States: Implications for Phenotypic Diversity

**DOI:** 10.1371/journal.pone.0011067

**Published:** 2010-06-10

**Authors:** Elena Shumay, Joanna S. Fowler, Nora D. Volkow

**Affiliations:** 1 Brookhaven National Laboratory, Medical Department, Upton, New York, United States of America; 2 National Institute on Drug Abuse, National Institutes of Health, Bethesda, Maryland, United States of America; Ludwig-Maximilians-Universität München, Germany

## Abstract

Human dopamine transporter gene (DAT1 or SLC6A3) has been associated with various brain-related diseases and behavioral traits and, as such, has been investigated intensely in experimental- and clinical-settings. However, the abundance of research data has not clarified the biological mechanism of DAT regulation; similarly, studies of DAT genotype-phenotype associations yielded inconsistent results. Hence, our understanding of the control of the DAT protein product is incomplete; having this knowledge is critical, since DAT plays the major role in the brain's dopaminergic circuitry. Accordingly, we reevaluated the genomic attributes of the SLC6A3 gene that might confer sensitivity to regulation, hypothesizing that its unique genomic characteristics might facilitate highly dynamic, region-specific DAT expression, so enabling multiple regulatory modes. Our comprehensive bioinformatic analyzes revealed very distinctive genomic characteristics of the SLC6A3, including high inter-individual variability of its sequence (897 SNPs, about 90 repeats and several CNVs spell out all abbreviations in abstract) and pronounced sensitivity to regulation by epigenetic mechanisms, as evident from the GC-bias composition (0.55) of the SLC6A3, and numerous intragenic CpG islands (27 CGIs). We propose that this unique combination of the genomic features and the regulatory attributes enables the differential expression of the DAT1 gene and fulfills seemingly contradictory demands to its regulation; that is, robustness of region-specific expression and functional dynamics.

## Introduction

Dopamine (DA) neurotransmission underlies core brain functions, including locomotion, behavior, cognition and motivation; consequently, disruption in dopamine signalling gives rise to various neuropsychiatric disorders and conditions i.e., Parkinson's disease, schizophrenia, attention deficit/hyperactivity disorder (ADHD) and addiction [1]. A key player in regulation of DA signalling is the dopamine transporter (DAT), it modulates the dynamics and the levels of DA in the synaptic cleft by recycling extracellular DA back into the presynaptic terminal. Alterations in the DAT availability in the brain directly affects the concentration of synaptic DA and the kinetics of its reuptake [2].

**Figure 1:**
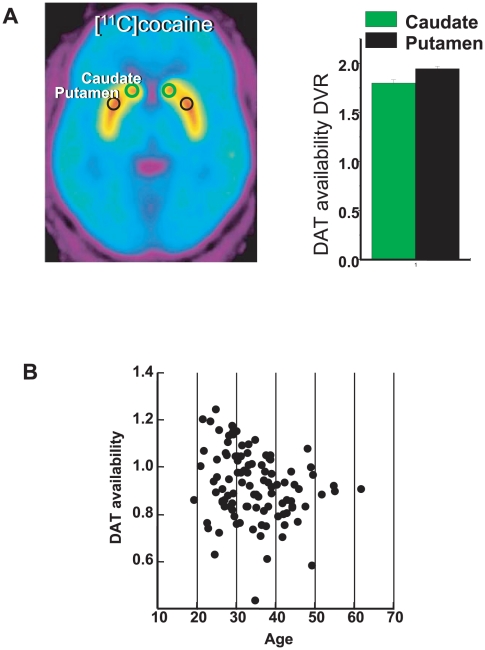
Variability of the dopamine transporter. **A** - DAT distribution in the brain. Averaged DVR [^11^C]cocaine image at the level of the striatum and ROI measures of DAT availability in caudate and putamen. Values represent means±standard deviations across 14 healthy men. **B** - DAT availability in the striatum of healthy controls of different ages. Measures were obtained with PET and [^11^C]cocaine and quantified as Bmax/Kd (using Logan plots for irreversible systems). Note the range of variation in DAT availability across individuals.

DAT expressed in the brain in a region-specific manner, most abundantly in the striatum (the mesostriatal DA pathway), where its concentration is more than ten fold higher than in the frontal regions (Figure 1A and [3]). Even in the same brain region, DAT expression significantly differs among individuals, reflecting high phenotypic heterogeneity (Figure 1B shows actual measures of the amount of the *DAT1* mRNAs in the midbrain; see also [4]). DAT expression is highly dynamic: the brain's DAT level adjusts to accommodate DA signaling, decreasing when DA release is low and increasing when DA release is high [5]. They change markedly in response to drugs [6], salient stimuli [6], environmental factors [7], and pathogens [8]. DAT expression alters throughout the life span; first detectable in developing brain relatively late, starting from 32 gestation weeks, the DAT level reaches maximum during adolescence, and then gradually decreases at an estimated rate of 5–6% per decade [9].

The possibility that the individual variations of basal DAT levels in the brain are due to genetic differences focused much attention on the *DAT1* gene (*SLC6A3*). It has been rigorously investigated in numerous clinical studies that aimed at revealing an association between the *DAT1* genotype and the risks for various brain-related diseases (reviewed in [7], [10]). By and large, these attempts had narrow scope: most focused on a single *DAT1* polymorphism, the functional 3′-UTR VNTR (variable number of tandem repeats) [11]. This polymorphism was associated with numerous neurological- and psychiatric disorders, including ADHD [7], cocaine-induced paranoia [12], tobacco smoking [13], and alcohol dependence [14], however, the findings have not been consistently replicated and most of the studies reported no effect of this polymorphism on neurophysiologic and psychiatric measures. Contribution of the 3′-UTR VNTR to differences in density and affinity of DAT was also investigated by several imaging studies; several studies provided some evidence of support [15], though the others did not [16].

There is increasing recognition of the role of environment in the etiology of neuropsychiatric disorders; in most instances such disorders arise through the interactions of genetic and environmental factors (reviewed in [17]). The environment is a potent genetic modifier, influencing gene expression via epigenetic mechanisms. Ubiquitous epigenetic mechanisms represent an essential element of normal development and maturation. Aberrant epigenetic processes can cause maladaptive changes, (gene dys-regulations and dys- functions) and consequently lead to disease. In the brain, in contrast to somatic tissues, epigenetic processes remain active throughout the lifespan: they ultimately are involved in the maintaining brain functions, enabling adaptive plasticity and the ability to accommodate varying environmental challenges [18]. The fidelity of epigenetic processes is critical for the human brain since its development creates enormously complex biological patterning. Consequently, it is most susceptible to aberrant activity of epigenetic modifiers: epigenetic dysregulation is implicated in pathogenesis of a variety of brain-related diseases, including mental retardation and complex psychiatric disorders [19].

The intricate pattern of DAT regulation points to its control by epigenetic mechanisms. Indeed, experimental and clinical studies support the concept of *DAT* gene-environment interaction. Data from animal models demonstrated that environmental insults during embryonic stage trigger changes in DAT level and delay the development of plasticity in the mesolimbic dopamine system [20] while in humans, early developmental exposure (*in utero* exposure to tobacco smoke) contributes to the risks and the pathogenesis of ADHD [7].

Currently many research groups are intent on unraveling the mechanisms regulating DAT availability in the brain and on identification of the genetic determinants that are involved in the *SLC6A3* control. Disparities in the results of traditional genetic studies of the *DAT1* genotype-phenotype relationship and evidence for gene-environment interaction call for a novel approach to studying the *SLC6A3* gene variations accounting for epigenetic mechanisms. We concentrated on developing a novel regulatory paradigm for the *SLC6A3*, wherein genetic- and epigenetic features are interconnected. Considering the facts about the human *DAT1* expression (summarized above), we hypothesize that it has unique genomic features that provide a basis for variable epigenetic sensitivity. Under this assumption, the individual variations in the *DAT1* gene sequence can directly affect its epigenetic potential, so that we might construe large inter-individual differences in brain's DAT level as phenotypic manifestations of the combinatorial interplay of the *DAT1* haplotype and epigenetic marks that jointly regulate the *DAT*. Empirical evidence supports our hypothesis; thus, the study of the effect of developmental exposure to tobacco smoke clearly demonstrated the risks of developing ADHD and the severity of the disease depends on the *DAT1* genotype [7]. Likewise, the *DAT1* genotype moderates the correlation between the risk for ADHD and early childhood adversity (institutional deprivation) [21].

To elaborate our hypothesis and to gain supporting theoretical evidence, we undertook a bioinformatic analysis of the *SLC6A3* locus using various genomic databases and computational analytical tools available via public domains (Table 1). Knowing that different software used to analyze same functional category are based on different algorithms and used different set of assumptions, and that random selection of one computational approach over another could bias results and introduce errors of interpretation, whenever possible, we applied several non-redundant programs in the same analysis. We selected the programs best suited to perform tasks and then ran them and compared their outputs to compile exhaustive results. If, however, the outputs obtained by different application were similar results, we choose a representative one to report in the manuscript. Our approach was to compare the *serotonin transporter* gene *(SERT or SLC6A4)*. The *SLC6A4* gene belongs to the same family of monoamine transporters and exhibits a high degree of sequence similarity with the *SLC6A3* (BLAST E-value = 2^e-166^); nevertheless, these genes have strikingly different expression patterns suggesting differences in their regulation. In addition, we considered that our comparative analysis would clarify whether the genomic features of the *SLC6A3* gene are truly unique or shared with phylogenetically related genes.

**Table 1:** Databases and Computational tools used for the analysis.

Functional modules	Genomic features	Toolkit
*Sequence variations*	SNPs	Genome Variation Server - http://gvs-p.gs.washington.edu/GVSNCBI dbSNP - http://www.ncbi.nlm.nih.gov/projects/SNP,
	Tandem repeats	UCSC Browser tools - http://www.genome.ucsc.eduTandem Repeat Finder - http://tandem.bu.edu/trf/SERV - http://www.igs.cnrs-mrs.fr/SERV/Mobile portal, e-tandem and geecee - http://mobyle.pasteur.fr/TandemSWAN - http://favorov.imb.ac.ru/swan/
	Copy number variations	Database of Genomic Variants - http://projects.tcag.ca/variation/
	Clusters of short interspersed repeats	REPFIND - http://zlab.bu.edu/repfind/
*Cis-and Trans-Regulatory regions*	Promoter prediction	Promoter 2.0 - http://www.cbs.dtu.dk/services/Promoter/Eponine TSS - http://www.sanger.ac.uk/Users/td2/eponine/CoreBoost_HM - http://rulai.cshl.edu/tools/CoreBoost_HM/
	Alternative promoter	PROSCAN - http://www-bimas.cit.nih.gov/molbio/proscan/PAZAR database - http://www.pazar.info/, ORCA toolkit
	Transcription Factor Binding Sites	CisRed - www.cisred.org/;JASPER - http://jaspar.cgb.ki.se/.Cluster-Buster - http://zlab.bu.edu/cluster-buster/.
	Sequence features	QGRS Mapper - http://bioinformatics.ramapo.edu/QGRS/analyze.php
	Nucleosome positioning	NXregions - UCSC Browser, Custom Track
*mRNA processing and stability*	Alternative splicing	SPLICY - http://host10.bioinfo3.ifom-ieo-campus.it/splicy/EBI ASTD - http://www.ebi.ac.uk/astd/main.htmlExonScan - http://genes.mit.edu/SpliceInfo - http://140.113.239.236/SpliceInfo
	3′-UTR secondary structure	GeneBee - http://darwin.nmsu.edu/~molb470/fall2003/Projects/samara/genebee.htmlSECentral Clone Manager Suite
	microRNA	mirBASE - http://microrna.sanger.ac.uk/sequencesTargetScan - http://www.targetscan.org/Pictar - http://pictar.mdc-berlin.de/EBI ASTD - http://www.ebi.ac.uk/astd/main.html

**Figure 2:**
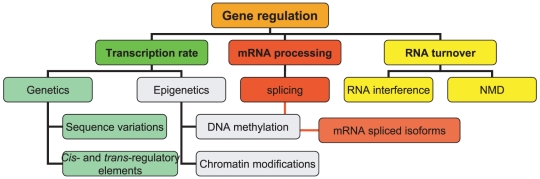
Gene expression regulatory mechanisms: schematic overview. Gene expression represents a multistep pathway that involves highly regulated and interconnected events; conceptually, it could be divided on transcription, mRNA processing and RNA turnover. Transcription rate is settled on genetic and epigenetic features of gene sequence; differential mRNA processing includes alternative splicing of transcripts, and RNA turnover rate which is determined by RNA stability depends on the RNA interference and nonsense mediated decay (NMD).

We first scrutinized in detail the gene sequences to identify various genomic characteristics may factor into expressional diversity. Next, we looked at different arms of gene regulatory network individually to identify which are involved in the *SLC6A3* regulation (Figure 2). Subsequently, we assessed a potential impact of the individual variations in the gene sequence on the gene's sensitivity to a particular regulatory mode and on its downstream phenotypic manifestations.

Our findings reported here deepen current understanding of the functional significance of the variations in the *DAT1* sequence. We provide evidence that the individual variations on a sequence level ultimately affect DAT's epigenetic sensitivity and the interplay of genetics and epigenetic mechanisms gives rise to wide diversity of dopaminergic phenotype. We identified new genetic targets; should experimental testing validate their biological significance, these targets potentially will have practical implication in advancing the development of treatment strategies and improving reliability of genetic screening of devastating diseases arising from impairments of the dopaminergic circuits.

## Results

### Conserved elements in the genetic loci

**Figure 3:**
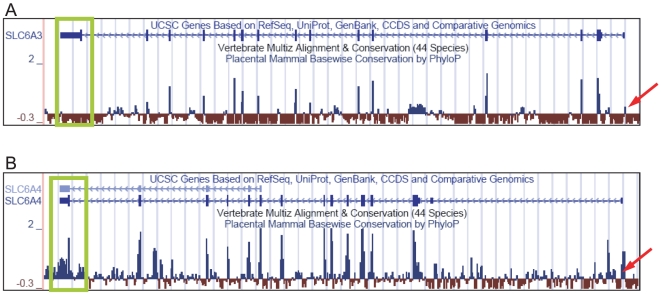
Conservation profiles of the *SLC6A3* and the *SLC6A4* genomic regions. Conservation tracks of the cognate genes loci shown in UCSC Genome Browser interface. A new Conservation track includes a 44-way vertebrate alignment; the subtracks display phyloP scores (in blue and red). Red arrow points to the promoter regions. The 3′-end of the genes framed in the green box. Note that the conservation of the regulatory regions across vertebrate species is evident only in the *SLC6A4* locus. In contrast, in the *SLC6A3* locus the only visibly prominent is the “negative” phyloP track, which score indicates accelerated evolution of the region.

**Figure 4:**
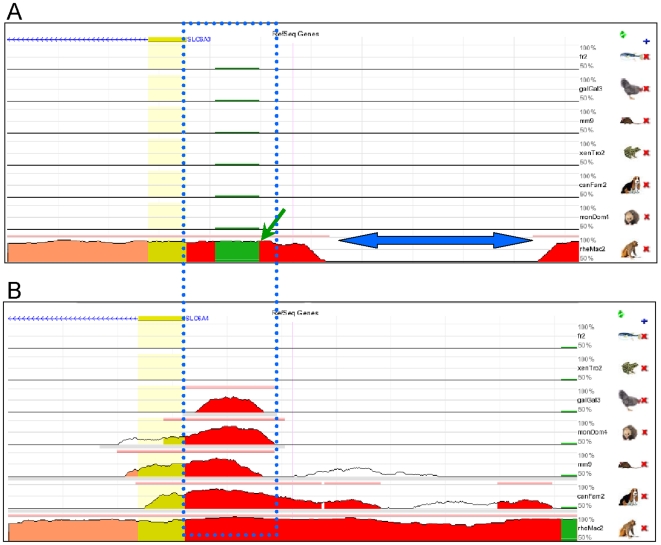
Conservation blocks in the 5′ regulatory regions of the *SLC6A3* and the *SLC6A4* genes. The ECR Browser was used to identify conserved regions within the *cis*-regulatory sequences of the *SLC6A3* and the *SLC6A4* genes. In the *SLC6A3* (**panel A**) sequence similarity of core promoter is limited to the *Pan troglodyte*; a low complexity repeat (indicated by green arrow) that resides in the core promoter of the human gene, lacks in the primate gene. The sequence of extended promoter presumably containing promoter enhancer elements (shown in red) is uniquely human as well (blue arrow). Core promoter of the *SLC6A4* is phylogenetically conserved across multiple species (**panel B**, blue box); its extended promoter is shared between humans and primates.

The protein composition of dopamine transporter is highly evolutionally conserved reflecting the fact that dopaminergic neurotransmission is an ancient signaling mechanism. However, conservation on the protein level is not reciprocal with conservation on the genome level. As is evident from the Figure 3A, sequence conservation of the *SLC6A3* locus across phylogenies at most is very limited: Sparse conserved elements are confined to coding sequences whilst the regulatory regions, including promoter, introns and 3′-UTR do not exhibit conservation (Vertebrate Multiz Alignment and Conservation, 44 species). Far more pronounced conservation is apparent in the *SLC6A4* locus with major conservation spikes corresponding to the coding regions, 5′ regulatory region and 3′-UTR (Figure 3B), suggesting that the other genes of monoamine transporter family are much more conserved. Phylogenetic analysis using other programs produced similar results, thus, our inspection of the entire *SLC6A3* and the *SLC6A4* loci (ORKA toolkit, PhastCons multi-species analysis) revealed only 3 conserved regions in the *SLC6A3* locus (including 5 kb flanks) versus 13 regions in the *SLC6A4*. Since evolutionary conservation commonly serves as a metric for identifying putative regulatory regions, the results of our analyses indicate that the regulatory mechanisms controlling the human *SLC6A3* evolved recently. Strikingly, even core promoter of the human *SLC6A3* (Figure 4 A) displays similarity with primate but not with other mammals where low complexity repeat (green) occur only in human genome. The *SLC6A4* core promoter is far more phylogenetically conserved (Figure 4 B, blue dotted box). Extended promoter region of the human *SLC6A3* is unique (Figure 4 A, blue arrow), while the sequences of extended promoter of the *SLC6A4* are similar in human and chimp (Figure 4 B).

The poor conservation of the *DAT1* locus might signify that the abundant changes in the sequence all are “effectively neutral”, that is, they do not affect fitness [22]. However, this is not the case because we noticed that the surrounding chromosomal regions do not exhibit such conservation dips. Accordingly, the changes in the *SLC6A3* sequence are rather locus-specific and can not be explained by isochore structure of the chromosomal region thus suggesting that the other evolutional forces drive this process, a bias gene conversion [23] for example. On the evolutional timescale, it appears that the major changes on the *DAT* sequence occur after a chromosomal translocation event during which the *SLC6A3* locus changed its genomic position from chromosome13 in rodents to chromosome5 in primates. Expectedly, the loss of the synteny of the genomic locus, which entails a very dissimilar chromosomal environment, adds to the phylogenetic divergence of the regulatory mechanisms of the *SLC6A3*.

Reconstruction of hominid evolutionary history revealed that the ancestral branch leading to primates and humans experienced a fourfold acceleration of segmental duplication accumulation whereas the rate of other types of genomic rearrangements, such as single-base-pair mutations, was far slower [24]. The *SLC6A3* locus is characterized by abundance of repeats and occurrence of chromosomal rearrangements (see below). Moreover, much of repeat polymorphisms in the *DAT1* sequence, including 3′UTR VNTR, is specific for human lineage and not shared even with primates, suggesting that these variations are positively selected. Interestingly, it is postulated that the sequences that are not shared between human and chimps may be important for human-specific traits [25].

### Sequence Variations

Much recent progress defining the molecular basis of phenotypic variability resulted from studying genomic DNA. Since this topic is extremely broad, in this analysis we focus on three major types of sequence variations, that is, single nucleotide polymorphisms, repeats and large-scale copy number variations. The surprising ubiquity of these variations in population greatly increases the spectrum of potential genetic variability, both enhancing the complexity of ‘normal’ genome and highlighting these forms of genetic variation as potential disease mechanisms.

#### Simple Nucleotide Polymorphisms (SNPs)

Point mutations in the DNA sequence (SNPs) are considered as major source of genome variations and are the best understood mechanism for phenotypic change [26]. Traditionally, genetic studies addressing such polymorphisms focused only on non-synonymous coding SNPs assuming that only these are functionally significant. Recently, it became apparent that so-called synonymous mutations are not neutral [27] but affect gene expression in several different ways. For example, SNPs occurring in 3′-UTR SNPs modulate the translation process by impacting mRNA transport [28], interference with the compartmentalized expression of tissue-specific genes in particular cellular loci [29] and altering mRNA stability [30]. Non-coding SNPs may trigger nonsense-mediated decay [31], inactivate the native splicing site [32], hinder posttranslational mRNA modifications [33]. Synonymous SNPs, while not affecting amino-acid composition of the cognate protein, may lessen accuracy and reduce translation rate [34], thereby leading to aberrant protein folding [35] because all these processes rest upon codon specificity.

**Figure 5:**
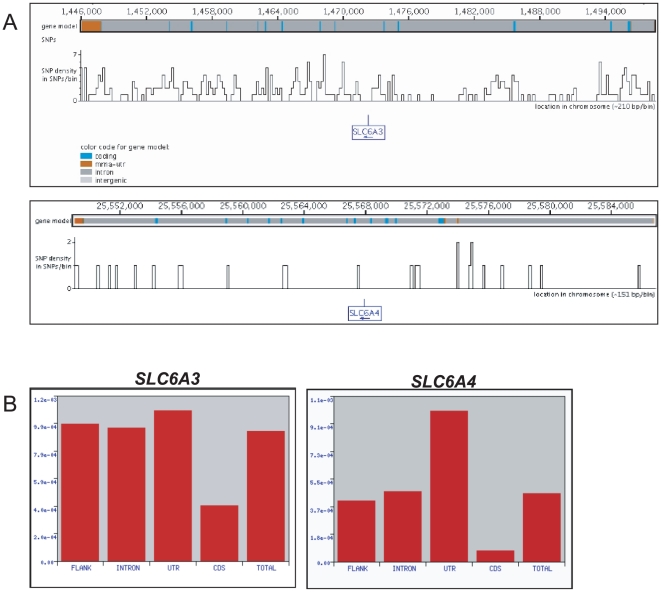
SNPs frequency and distribution within the chromosomal regions of the *SLC6A3* and the *SLC6A4 genes*. **A –** Chromosomal maps were produced by the Genome Variation Server in a “summary SNPs display” view (the *SLC6A3* map - top panel, and the *SLC6A4* map – the lower panel). Single peaks are indicating genomic positions of SNPs where peaks height corresponds to the SNP frequency (filtering – default options, merge option *- common samples with combined variations*). **B -** Regional frequency of the SNPs in the *SLC6A3* (left) and the *SLC6A4* (right).

Multiple SNPs are mapped to the *SLC6A3* locus: The NCBI SNP database (frozen April 7, 2009) lists **897** of them. This number significantly exceeds the number of SNPs in the other genes of the monoamine transporters family; thus the *SLC6A4* gene has 441 SNPs and the *SLC6A2* (norepinephrine transporter gene) has 514 SNPs (Figure 5A). Not only the numbers, but also the patterns of the SNPs distribution in the *SLC6A3* and in the *SLC6A4* loci are markedly distinclt: SNPs are found in virtually all *SLC6A3* domains (flanking regions, coding sequences, exons and introns), whereas in the *SLC6A4*, they largely are confined to the UTR region (Figure 5B).

The thirty-three SNPs mapped to the *SLC6A3* 3′-UTR potentially can hinder localized translation of the *DAT1*. We noted (GVS) that the 3′UTR SNPs have low conservation scores (mostly 0.000), indicating an evolutionally recent origin, so they likely are human-specific. Considering that five 3′UTR SNPs are common in human populations, with minor allele frequencies of 16% and higher (Table S1 lists the SNPs), they might add a factor to the variation in *DAT1* expressional. Interestingly, two SNPs in the *SLC6A4* 3′UTR have high conservation score (0.99 and 0.98), suggesting that they occur in different species.

The 5′ gene region is pivotal for regulation, it encompasses binding sites for transcription factors and components of basic transcriptional machinery; consequently, it is unsurprising that 5′UTR often is highly conserved and rarely carries variations. We found that the 5′ regions of the *SLC6A3* and the *SLC6A4* genes have 31 and 12 SNPs, respectively, suggesting that they might confer expressional variability.

Below, we elaborate on possible functional consequences of SNPs in regards to their potential impact on particular regulatory mechanisms.

#### DNA repeats

Repeated DNA sequences are common feature of the human genome (209,214 loci, according to [36]). Organized in tandem DNA repeats (tandem repeats) are inherently unstable, and known to be the “hotspots” for mutations [37]. However, their abundance in the human genome posits that they may not be strictly deleterious but might confer some evolutionary advantage, such as affording transcriptional divergence, for example [38].

The 3′ UTR VNTR of the *SLC6A3* is the most studied polymorphism in this gene and has been linked to variety of clinical conditions [39]. The VNTR in the intron8 of the *DAT1* was also explored in clinical- and preclinical settings and was linked to cocaine abuse [40] and smoking cessation [41]. The high SNPs frequency in the *SLC6A3* gene led us search for possible repeat variations in its sequence. We analyzed the current version of the *SLC6A3* sequence (Human 2009 Assembly (hg19) running several *ab initio* prediction programs. We repeated the same analysis with different software and found that even thought the outputs were different in actual numbers of the repeats, the results were consistent. The Tandem Repeats Finder (TRF 4.04) [42] that uses conservative approach, detected 71 repeats, the e-tandem identified 30 regions and the TandemSWAN [43], designed to identify imperfect or “fuzzy” repeats generated more than 600 hits.

Intrigued by the high number of repeats, we wanted to define some principal parameters associated with tandem repeat polymorphism in order to predict their variability (or hypervariability). G + C content of the sequence is a strong predictor of minisatellite polymorphisms [44], and, since the *SLC6A3* sequence composition is CG bias (0.55, geecee of Mobyle Portal) it is likely that at least some of newly predicted repeats are polymorphic. The SERV software [45] assessed the polymorphic potential of the predicted repeats by assigning the score to the repeat region (VARscore). The score value between 1 and 3 was proposed as an indicator of potentially good genotyping target, and it was found that the VARscore and experimentally determined polymorphisms are in good correlation [45]. Seven of the *SLC6A3* repeats had the optimal score thus representing potential new genotyping targets (Table S2). We noted that the VARscore 2.31 was assigned to the 3′UTR VNTR region; this fact strengthens the validity of our computation. It is noteworthy that the sequences of the longer tandem repeats in the *SLC6A3* locus often itself contain simple repeats. Simple repeats, especially trinucleotide repeats such as CAG, CTG, CGG, are the most unstable and often are associated with human diseases.

We used the same methodology to analyze the *SLC6A4* gene: in this sequence the SERV detected 40 repeats, the Mobile portal found 11 and the TRF found 30 repeats. In contrast to the *SLC6A3*, the repeats in the *SLC6A4* have shorter consensuses overall and only three repeats have a period longer than 30 bp (Table S3). For the potentially novel genotyping targets, we might consider only one region (VARscore 1.36). Of notice, analyses were limited to the gene sequences; therefore the region of the 5-HTTLPR polymorphism was outside the scope.

#### Copy number variations (CNVs)

Copy number variations refer to the incidence of additional or missing chromosomal segments in some individuals, adding another dimension of variation to the genome [46]. This genomic feature is highly prevalent: about 13% of human genes overlap with CNVs [47]. CNVs are significantly overrepresented in telomere-proximal regions and in simple tandem repeat sequences; they often detected in genes that have significantly elevated nucleotide substitution rates. The non-uniform distribution of CNVs in the human genome suggests that they have functional consequences; according to Fraser and others, [48], CNV's contribution to genetic diversity might exceed the combined contributions of all other genetic variations. As thought, the CNVs might help to explain some cases of diseases with evident genetic causation but lack of a genetic clarification [49].

Multiple segmental duplication in human genome are recent and a large fraction of these regions is not duplicated in the chimpanzee (Pan troglodytes) genome [50]. Of direct relevance to the subject of our analysis is the fact that the localization of these human-specific duplications is bias for chromosomes 5 and 15 [51].

Our inspection of the *SLC6A3* locus (DGV) revealed three structural variants mapped within the gene body (Figure S 1. Note that the largest CNV (36355) involves two exons – exon 5 and exon 6). However, that the actual number of the CNVs in the *SLC6A3* locus might be much larger; indeed, the annotations to the latest release of Human Genome Assembly (hg19, 2009, UCSC) include references to the multiple deletions of different sizes, including a deletion of 489 nucleotides (dbSNP build 130 rs71309291). Considering that CNVs data are obtained by analyzing genomic DNA from healthy individuals [46], the CNVs detected in the *DAT1* sequence have no apparent phenotypic consequences or have only subtle effects. Alternatively, these findings might be limited to a particular subpopulation of the blood cells (mosaicism) that arise due to the inherent genomic instability of the *SLC6A3* locus. The high rate of the CNV occurrence in the *DAT1* locus (three out of eight individual genomes tested revealed CNVs in the *DAT1* gene), indicates that the copy number variations encompassing the *DAT1* locus are frequent in the normal population and, therefore, may be advantageous. Lack of CNVs in the *SLC6A4* and the *SLC6A2* loci suggests that, in contrast to the *SLC6A3*, they are controlled by purifying selection.

### Transcriptional regulation

#### Inspection of the Promoter region

Our next step was to explore the promoter region of the *SLC6A3*. Experimental and clinical evidence indicates that sequence variations upstream of the transcription start site (TSS) affect *DAT1* transcriptional regulation [52]; therefore, we evaluated the promoter region in greater details. A promoter sequence represents a template recognized by the transcriptional machinery; therefore promoter analysis is essential for understanding transcriptional regulation of the cognate gene. It is difficult to accurate locate the position of the TSS and to exactly specify the promoter region because the sequences of the human promoters have exceedingly diverse characteristics.

Since the *SLC6A3* core promoter lacks “TATA” and “CAT” boxes (these DNA sequences provide docking sites for basal transcriptional complex) [53], we must identify other regulatory sequence elements that could substituting for them. Transcriptional initiation of neuronal genes often is facilitated via binding of core transcriptional machinery to the CCAAT element [54]; not unexpectedly, we detected several CCAAT consensuses upstream and downstream of the *SLC6A3* TSS suggesting that the *SLC6A3* might be one of these. Human genes that have CCAAT-promoters display several common characteristics; in general, they are less precise in terms of TSS then the genes with TATA-promoters and they mostly overlap with CpG islands. Transcription initiation from the CCAAT box involves NF-Y, an element with histone-like features, and a particular subset of transcription factors [55]. Both features are relevant to the epigenetic sensitivity of the *SLC6A3* as we elaborate later.

Comparing the regulatory regions of the *SLC6A3* and the *SLC6A4* revealed their markedly different regulatory potential. While regulatory region of the *SLC6A4* displays characteristics of a strong promoter (high significance of predicted TSS), the 5′ region of the *SLC6A3* does not display the typical sequence characteristics of a strong promoter. Here, *de novo* predicted TSSs are only marginal (Promoter 2.0 Prediction server) or off-target (Softberry). The software Eponine (Sanger) predicts multiple low-score (ambiquious) TSS sites are predicted by the Eponine, the CorePromoter [56] denotes ten putative TSSs with comparable low scores and the AceView [57] predicts three putative alternative promoters for the *SLC6A3* gene.

The complex structure of human gene promoters with a range of alternative transcription start sites (TSSs) [58] supports differential temporal- and spatial- patterns of gene expression and provide an additional level of gene regulation by modulating translational efficiency [59]. About one fifth of human genes have alternative promoters; this phenomena is most frequent in brain-related genes [60]. Based on our analysis we might categorize the *SLC6A3* promoter as a broad one; in addition, absence of a strong promoter indicates the potential for off-target transcription initiation.

The heterogeneous dopaminergic neurons within the mesocorticolimbic dopamine system have distinct anatomical and physiological properties [61], therefore, the biological identity of a specific subpopulation of dopaminergic neurons might be enabled by preferential transcription of particular mRNA. Our computational prediction that the *SLC6A3* transcription might lead to production of different mRNA isoforms is strengthened by *in vivo-*derived evidence: the *SLC6A3* transcripts of different length were detected in different brain regions (long transcript M95167 in the brain stem [62], and short transcript, L24178, in *Substantia Nigra* [63]), suggesting that alternative transcription initiation might enable region-specific expression of the *SLC6A3*. Furthermore, differential transcription initiation might be a means for a rapid switch in response to the acute environmental cues: Prefolded RNA structures are less efficient in adopting environmental changes into expressional changes [64], [65], which might be explained as reflecting the high thermodynamic barriers to interconversion between the secondary structure isoforms.

Whether the *SLC6A4* regulation involves similar regulatory mode remains unclear since almost all RefSeq annotated human *SLC6A4* transcripts are of somatic origin (placenta, lung) and of similar length. However, it is likely that it, indeed, utilizes AS, given that the rat *Sert* gene has two alternative promoters and its transcripts are alternatively spliced in a tissue-specific manner [66].

#### Transcription factor binding sites (TFBSs)

The interaction of the *cis*-regulatory elements of a gene with transcription factors (TF) largely determined transcription event, therefore, an assessment of the putative TF binding sites in the regulatory region of the gene under analysis yields important information on this gene's regulation. In the nervous system, TFs define the basic framework [67]; their availability varies across the brain regions and cell types thus contributing to the phenotypic diversity [67];. As we mentioned, the nature of the *SLC6A3* promoter (CCAAT promoter) implies its sensitivity to selective TFs, because NF-Y synergistically interacts with a subset of TFs [55]. Our inspection of the *SLC6A3* 5′-flanking sequence (−2 kb, JASPAR database) revealed that it contains binding sites for Sp1, GATA-1, CREB, and c-Myc cis-acting regulatory elements –all those TFs interact with NF-Y [55]. The Sp1-sensitivity of the DAT transcription was previously demonstrated *in vitro* [68], thus validating the biological significance of the sites we detected. Sp1 is a key element of non-classical genomic nuclear receptor pathways mediating hormone-dependent gene activation and repression suggesting the possibility of the involvement of a hormonal element in *DAT* regulation.

The nucleotide sequences recognized by individual TFs are too small and degenerate to enable detection of functional enhancer and silencer elements within the genomic context. Therefore, it was postulated that dense clustering of the motifs may improve the accuracy of their recognition and enable better control of the gene expression level [69]. Using the Cluster-Buster web server [69] to query promoter sequences of the *SLC6A3* and *SLC6A4* genes, we found a high-scoring (9.15) cluster in the *SLC6A4* but not in the *SLC6A3*. Next, we compared the TFs profiles of both genes (CisRED [70]) noting their distinctions (Figure S2). While the lengths of the cisRED regions are comparable, they differ in their architecture: The atomic TF motifs are densely clustered in the vicinity of the *SLC6A4* TSS, but and rather scattered in the *SLC6A3* regulatory region. Collectively, these results suggest that TFs-mediated transcription more important for the *SLC6A4* regulation than for that of the *SLC6A3*.

#### Quadruplex forming G-rich sequences in pre-mRNAs and mRNAs (QGRS)

The unusually frequent occurrence of CG nucleotides in the *SLC6A3* sequence (CG content 0.55 compare to 0.47 in the *SLC6A4* sequence) likely influences the physical properties of the DNA molecule, since guanine-rich DNA tends to adopt a four-stranded structural form known as a G-quadruplex, or G4 DNA [71]. QGRS are highly conservative and often found in key chromosomal regions, suggesting that they have functional significance. When located in the promoter region, G-quadruplex structures can act as cis-regulatory elements and either enhance transcription of, or silence the respective gene. Apparently, natural selection progressively favored the canonical G4 DNA motifs within regulatory regions and, consequently, G4-formation has been proposed as a mechanism for regulation of gene expression in eukaryotes [71]. CG-rich mRNAs also form highly stable G-quadruplex structures [72], which play major role in mRNA turnover [73] and translation [74].

To gain an insight on the involvement of G4 DNA formations in the regulation of the *SLC6A3*, we analyzed the landscape of G4 DNA (PG4Ms) motifs in the human *SLC6A3* gene using the GRS database [73] with the default settings and filters of the server. Unexpectedly, we found that the multiple G4 motifs that are not restricted to the TSS-proximal regions. The number of non-overlapping QGRS in the *SLC6A3* sequence (474) almost doubles the number of G4s in the *SLC6A4* and the *SLC6A2* sequences (203 and 281, respectively). This high score of detected QGRSs increases the likelihood that G-quadruplex structures form *in vivo*, thus, adding another factor to the *SLC6A3* regulation. Importantly, several QGRS were detected in the vicinity of the intron-exon junctions of the *SLC6A3*: Because G-rich quadruplexes are actively involved in alternative and tissue-specific regulated splicing events [75], their positional effect on gene expression and the implication for DAT function could be most profound.

### Post-transcriptional regulation

Post-transcriptional level of gene regulation comprises a series of post-transcriptional and -translational mechanisms that increases the complement of neuronal transcripts and protein isoforms [76]. Neuronal mRNAs are diversified through several processes including alternative splicing, compartmentalized translation, and RNA editing. In most tissues, transcription-generated RNA base sequences experience downstream alterations; this ubiquitous process is particularly active in the nervous system [77].

#### Short interspersed repeats

One of the mechanisms of gene regulation involves controlled transport of the mRNA to the subcellular compartment where the gene's protein products are used. The half-life of a particular mRNA depends in part on the sequence of the 3′ UTR of the transcript such as clusters of short interspersed repeats (4–7 bp) that can regulate alternative splicing and mRNA stability [78]. We identified more that fifty short repeated units in the 3′- UTR of the *SLC6A3* (about 2 kb) (REPFIND [79]) with a P-value less than 1.11^e-16^, signifying that they are not random but likely to be of functional significance (Figure S 3A). A similar analysis of 3′-UTR of the *SLC6A4* revealed only ten short repeated units, with weaker significance where the smallest of P- values, assigned to one of the repeats, was 7.78^e-06^. We noted that short interspersed repeats are scattered across the 3′ region of the *SLC6A4*, whereas in the *SLC6A3* gene, they are largely confined to the middle of the 3′-UTR. This is an important fact, since this positional overlap suggests that the length of the polymorphism directly affects the number of the short interspersed repeats.

In neurons, effective mRNA translation occurs near dendritic terminals [80], [81]. The binding of specific proteins to cognate mRNA sequences and formation of translocationally-competent nucleoprotein complexes enables the intracellular trafficking of mRNA molecules. The proteins of translocation machinery recognize CAG or CAG-containing repeated motifs in the 3′-UTRs of the mRNA molecule; such short motifs are commonly detected in genes that are translated in dendrites [79], [82]. Assuming that the *SLC6A3* is one of such genes, and its translation likely occurs near synaptic terminals, we screened its 3′ UTR for CAG motifs; as expected, we found multiple such tri-nucleotides repeats and repeats clusters (Figure S3B). Surprisingly, we did not detect CAG motifs in the 3′ UTR of the *SLC6A4*. The basis for this difference is unclear, since in applying the same reasoning, *SLC6A4* translation in neurons is likely to take place near synaptic terminals. Possibly, the lack of a translocation signal is due to the abundant expression of the *SLC6A4* in non-neuronal cells. Alternatively, the intracellular translocation machinery might recognize the sites within the coding sequence of the *SLC6A4* mRNAs, as was established for the dendritic trafficking of the *BDNF* transcripts [83]. Notably, the short interspersed repeats and the 3′-UTR VNTR polymorphism of the *SLC6A3* reside in the same region, suggesting the complexity of possible biological effects of sequence variations occurring therein. We will discuss this issue in more detail later.

#### Splicing and Alternative splicing

Because pre-messenger RNA of contain multiple introns, the splicing is a critical step in its expression. Alternative splicing (AS) generates multiple splicing isoforms with different combination of exons thus dramatically increasing the molecular complexity of the expressed proteins [84]. The AS rate progressively increased during evolution: in yeast only a small portion of genes is alternatively spliced, while in the human genome, the majority of protein-coding genes are known to undergo alternative splicing [85], [86], [87]. The production of differential isoform varies across tissues and cells: the rate of AS events and the level of alternatively spliced mRNA in the brain are much higher than in somatic tissues [88], [89].

We searched for evidence supporting the involvement of AS in the *SLC6A3* regulation. Both, the *SLC6A3* and the *SLC6A4* genes have 15 exons, viz., more than the average of 9 exons for a human gene; and genes with higher number of introns and exons more often utilize AS. We also found a) there are at least three different mRNAs transcribed from this locus (AceView); b) the *SLC6A3*-mRNA L241781 encodes 11 exons (exon skipping, SliceInfo); and, c) two plausible intron/exon structures for the *SLC6A3* mRNA containing 15 and 16 exons (SPLICY database) as evident from microarray experiment data (probeset annotations). In addition, an ambiguity in position mapping for the first exon of the *SLC6A3* (First EF) and the number of *ab initio* predicted exons (26, as by the ExonScan) corroborate the possibility of AS of the *SLC6A3* transcripts.

The splicing pattern might be dynamically regulated by external stimuli; in fact, most cellular signaling pathways that change gene transcription also alter groups of alternative exons [90]. Neuronal activity and drugs can induce alternative pre-mRNA splicing [77], [91], [92]. For example, cocaine and dopamine trigger the synthesis of the ania-6 (cyclins protein family) in the brain, whereby, depending on the nature of the stimulus, AS generates two distinct ania-6 mRNAs that then are translated to functionally different proteins [93].

Fox-1 and Fox-2 proteins were identified as regulators of the neuron-specific splicing pattern [94] [95]. They contain a single highly conserved RNA-binding domain acting as a splicing enhancer in the intron downstream from many exons, and might induce exon skipping [95]. Fox proteins mediate splicing of many genes critical for neuronal development and brain function [96]. We found multiple high-scoring binding sites for Fox2 family proteins on the *SLC6A3* and *SLC6A4* transcripts suggesting they may represent an AS regulatory element.

Splicing machinery recognizes flanking sequences around the exon-intron junctions (∼20 bp); they are pivotal for the fidelity of the splicing process. Mutations within the splice sites of their auxiliary elements can either affect the splice site's efficiency abolishing splicing or alter the balance of the alternatively spliced forms, so modulating the phenotypes of the cognate gene's [97], [98]. The functional consequences of SNPs in the exon-intron junctions are as profound as those for SNPs in the coding regions [99], [100]. Splicing errors are implicated in pathogenesis of various diseases, including neurological diseases and malfunctions [101]. Strict selection control of exonic splicing elements explains the rarity of SNPs near intron-exon boundaries [102]. Contrary to our expectation, we found twenty- five DAT''s SNPs and eleven SERT's SNPs in the exon-intron junctions (Galaxy Browser). Apparently, these SNPs increase a probability of splicing errors, thereby engendering aberrant transcripts.

#### MicroRNA (miRNA)

A miRNA-mediated mechanism that destabilizes transcripts regulates gene expression at the level of translation (reviewed in [103]). MiRNAs play a primary role in controlling gene expression during development but also in post-mitotic cells, such as neurons, and apparently contribute to age-dependent variation in gene expression [104], [105]. While repressing target genes in actively proliferating cells, MiRNAs can activate gene expression in non-dividing cells such as neurons [106]. The sites of miRNA recognition often are found in the 3′UTR region [107]. To retrieve information on miRNA binding sites for the *SLC6A3* and the *SLC6A4* sequences we used the MirBase Target Database software (Welcome Trust), which recognizes 14 and 57 targets, while EBI ASTD, recognizes 107 and 46 targets, respectively. The identification of multiple targets supports the possible involvement of the miRNA machinery in regulating both genes.

Recently, our understanding of the mechanisms of miRNA-mediated gene regulation was broadened due to the discovery that human-specific miRNAs interact with sites in the 5′-UTR motifs [108]. An important sophisticated facet of this work was the finding that the miRNAs of this novel class interact simultaneously with both 3′ and 5′ UTRs targets; this “double” targeting greatly enhances miRNA's ability to down-regulate the gene expression. We found that the *SLC6A3* and the *SLC6A4* -genes are amongst the targets for this novel class of miRNAs (miBridge, [108]). In particular, the *SLC6A3* UTRs contain consensus sequences recognizable by eight miBridge' miRNAs, while the *SLC6A4* UTRs contain consensuses for 4 miRNA of this class. The target prediction algorithm implemented in the miBridge is more stringent than those in other software, and consequently, the number of genes predicted as miRNA targets by miBridge is low; however, the multiple identification of the *SLC6A3* gene as a target underscores the importance of miRNA-mediated control in its regulation.

#### Differences in conformational structure of SLC6A3 transcripts and their stability offer a putative mechanism explaining biological significance of the 3′VNTR polymorphism

The half-life of a particular mRNA depends in part on the sequence of the 3′ UTR that can regulate alternative splicing, the intrinsic stability of a cognate mRNA and its sensitivity to regulation by miRNA [78]. Transcript length, however, also determines the RNA's secondary structure; hence, various *SLC6A3* transcripts might fold differently. The conformation of the RNA molecule is essential for its biological function; it determines the translational rate and the biological catalysis of a transcript [109], [110]. We determined that the length of the *SLC6A3* mRNAs correlates with the stability of their compacted structures (results in Table 2), suggesting that there is yet another regulatory mechanism for the *DAT* gene that might operate via preferential transcription of one mRNA over another. More specifically, cells might employ a rapid switch in transcriptional mode to achieve fast increase in DAT level, hence, rapid synthesis of DAT protein; whereas the production of longer, more stable transcripts prolongs translation time determining basal DAT's level.

**Table 2:** The length of the *SLC6A3* mRNAs correlates with the stability of their compacted structures.

mRNA	mRNA length	Free Energy of assembly	Ensemble Diversity
**M95167.1**	**3946 nt**	**−1716.51 kcal/mol.**	**792.13**
**S46955.1**	**3919 nt**	**−1698.40 kcal/mol.**	**960.43**
BC132977.1	2774 nt	−1176.54 kcal/mol.	1032.98.
BC133003.1	2774 nt	−1176.54 kcal/mol.	1032.98.
**S44626.1**	**2020 nt**	**−868.85 kcal/mol.**	**678.67**
**L24178.1**	**2010 nt**	**−867.45 kcal/mol.**	**427.14.**

Note that increase in the mRNA length correlates with corresponding increase in free energy of secondary structure. Highest Ensemble diversity is predicted for the transcript isoforms of a medium length.

The 3′UTR VNTR polymorphism of the *SLC6A3* apparently changes the transcript length; therefore we sought that these differences might be a factor contributing to the phenotypic differences ascribed to the particular alleles. Following the original discovery of this polymorphism [111], there have been many experimentally and clinical investigations. The finding that the number of the tandem repeats affects the transcriptional efficacy of the *DAT* was confirmed by different experimental means but failed to provide satisfactory explanation to tshe phenotypic differences observed *in vivo*. By definition, 3′UTR variation does not affect the protein product; hence, it ought to exert its influence on the level of transcripts. We hypothesized that the actual number of the repeated consensuses in the cognate allele would determine the secondary structure of the corresponding *SLC6A3* transcript. We tested our hypothesis by modeling the secondary structures of putative mRNAs containing 9 versus 10 repeats, which correspond to the most common alleles in the population (GeneBee server [112]. We found a notable difference in the free energy of the predicted secondary structures of the respective mRNAs, viz., −135 kcal per mol (10 repeats), versus −95 kcal per mol (9 repeats) (Figure S4A). Further, we noted that the folding structures of mRNAs i.e., the tendency towards hairpin formation also are subordinate to the repeats number: Duplexes formed by the 9-repeat sequences had longer stems and higher energy than those formed by the 10-repeat sequences (−14.4 kcals versus 7.4 kcals) (SECentral Clone Manager Suite 7 program) (Figure S 4B). In sum, our analyses revealed that the number of the 3′ UTR repeats might affect the *SLC6A3* expression in several different ways; specifically, by transforming the secondary structure of the cognate transcript and modulating its propensity to form hairpin structures. The conformational differences of the mRNAs (determined by the transcripts lengths) might tangentially make an impact on other regulatory mechanisms, including the number of putative miRNA target sites facing the surface of the molecule could vary, changing the transcript's degradation rate. In a similar fashion, the colocalization of the 3′UTR VNTR region with the region of short interspersed repeats points to their co-regulation.

### Epigenetic regulation of the *SLC6A3* and the *SLC6A4* genes

Several studies have analyzed known polymorphisms in the *DAT* and the *SERT* genes as a function of specific environmental factors [113] proving that adverse environments entail disordered behavioral manifestations more often in the carriers of particular polymorphisms. There is a common agreement that the environmental factors play a role in the pathogenesis of brain disorders, but the mechanisms of gene-environment interactions on molecular level remain poorly understood. Our knowledge on the epigenetic status and dynamics of epigenetic processes regulating the *SLC6A3* and the *SLC6A4* is very limited due to significant technological challenges; therefore, systematic identification of possible targets amenable to epigenetic modifications by computational means intrinsically is worthwhile. Further, since a computational analysis is sequence-based, it is not influenced by the innate variability of experimental conditions.

#### DNA methylation

DNA methylation in the mammalian genome predominantly occurs on cytosine in context of the 5′-CpG-3′ dinucleotides; this is the only type of epigenetic modifications to directly change the DNA molecule. Stretches of GC-rich sequences in the genome called CpG islands (CGIs) that are associated with open transcriptionally competent chromatin structure were discovered in gene promoters [114]. The relevance of GC content and CpG dinucleotide concentration to the regulation of gene activity points to its physiological significance. The methylation status of the promoter-overlapping CpG island plays a central role in the epigenetic control of gene expression thus, aberrant methylation patterns (hypo- and hyper-methylation) can engender gene dys-regulation. Conceptually, an alteration in the pattern of DNA methylation might serve as an efficient mechanism for genes to adapt functionality in response to a variable environment and also might compensate for the effects of a mal-functional polymorphism. The DNA methylation pattern established during the development and differentiation is preserved with high fidelity during cell division by DNA methyltransferases (DNMTs). DNMTs are highly expressed in developing tissues but their activity declines during differentiation in all tissues except the brain wherein they are expressed throughout the lifetime in the brain [115], [116]. Dynamic DNMT activity in the brain is essential for synaptic plasticity and memory formation [48], [117].

**Figure 6:**
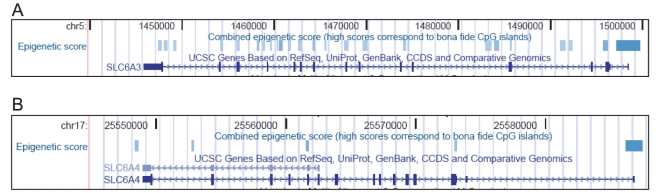
*Bona fide* CGIs in the *SLC6A3* and the *SLC6A4* regions. Visual displays of the bona fide CGIs (CpG islands) are obtained using Custom Tracks option (UCSC Genome Browser). Here, blue boxes represent predicted CGIs based on combined epigenetic score. Note, that in the *SLC6A3* locus (top panel), multiple GCIs are distributed within all gene regions whereas in the *SLC6A4* locus (lower panel), CGIs are evidently less numerous.

Because CpGs act as a mutation hotspot (deamination of methylated CpGs to TpGs) and the estimated mutation rates at CpG sites are about 10–50 times higher than other transitional mutations, CpG dinucleotides are under-represented in mammalian genomes where they occur at one-fifth of their expected frequency [118]. The human *SLC6A3* gene is remarkably GC dense and has multiple CpG islands: in contrast to most vertebrate genes that have only a promoter-overlapping CpG island, all gene regions of the *SLC6A3* display characteristics attributed to this genomic feature. We mapped twenty-seven *bona fide* CpG islands [119] to the human *SLC6A3* locus (Figure 6). Notably, even the “conventional” promoter-overlapping CpG island of the *SLC6A3* is unusual: on average, in active promoters of human genes cytosine and guanine account for 57% of the nucleotides [120] GCs represent 79% of the *SLC6A3* promoter sequence. The frequency of the intronic CGIs is approximately four-times lower than the expected value [121], yet we found 21 such CGIs in the *SLC6A3*. The *SLC6A4* locus contains six CGIs, which is also above average but lower than that for *SLC6A3* (Figure 6, bottom panel).

CGIs associated with genes underwent gains and losses during vertebrate evolution [122]. Human genes generally contain more promoter-associated CGIs and have more pronounced CGI characteristics [123] than do mouse genes. Seemingly, the number of CpG islands in the *DAT gene* dramatically increased during evolution: The mouse *Slc6a3* has no CGIs, the rat's gene has one promoter-associated CGI, while the rhesus monkey's *DAT* gene has two CGIs (one in the promoter and one intragenic). It is tempting to speculate that the increase in the number of CGIs in the *DAT gene* could be evolution-driven and reflects the growing demands dictated by higher brain function in humans.

#### Chromatin organization, Nucleosomes

*In vivo*, the DNA molecule forms a complex with proteins that allows its packaging into chromatin. Nucleosomes are the structural units of chromatin represented by histone octamers around which the DNA coils. The close interaction of the DNA molecule with a nucleosome core results in a condensed chromatin that is inaccessible to the transcription machinery; hence, the transcriptional activation of a gene requires the local transition of compact chromatin domains into decondensed loops. Nucleosome remodeling and covalent modifications of histones provide the basis for epigenetic gene regulation that occur via the modulation of the accessibility of the genomic loci to transcriptional machinery [124]. Dynamic chromatin- remodeling events largely determine the frequency of promoter activation, thus, the expression of many genes could be mapped to chromatin modifiers, rather than to TFs [125].

**Figure 7:**
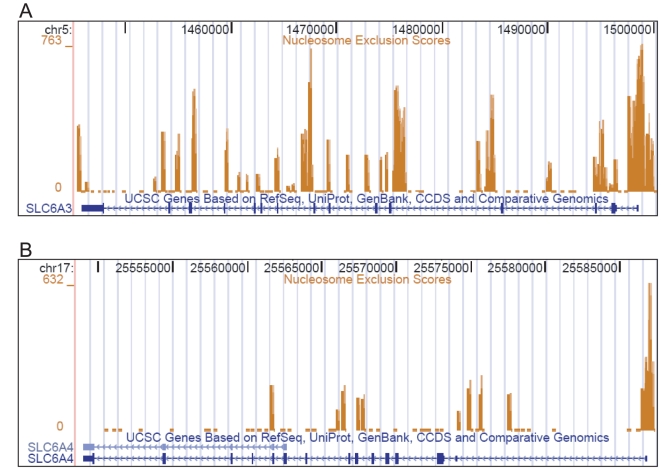
Nucleosome exclusion regions. Nucleosome-exclusion regions (NXRegions) are shown for the *SLC6A3* (top panel) and the *SLC6A4* (lower panel) genes. The figure was prepared by uploading NXScore results as a Custom Track on the UCSC Genome Browser interface where the snapshots showing the Known Genes track and Custom Track option were taken for the cognate gene's loci. NXScore peaks in the *SLC6A4* are largely confined to the TSS vicinity (as it is seen in most of the human genes) and the height of the TSS-overlapping peaks is dominant. Distinctly different pattern of the nucleosomes is predicted for the chromosomal region corresponding to the *SLC6A3*: here multiple NXScore peaks are densely mapped to the different regions of the gene body and to the flanks. Notice that the height of the TSS-proximal NXScore peak is comparable with the other intragenic peaks.

CG-rich motifs in DNA sequences inherently disfavor nucleosomes, and are referred to as “nucleosome exclusion sequences” (NX) [126]. We found that both, the *SLC6A3* and the *SLC6A4* genes have high NX-scoring sequences near the TSS (Figure 7). The predicted nucleosome positioning in the *SLC6A3* and the *SLC6A4* loci notably differ: Entire *SLC6A3* locus comprises of numerous nucleosome-dysfavouring sequences, while in the *SLC6A4*, NX-peaks are sparse (Figure 7, top and bottom panels). It was suggested that intragenic regions with high NXScores might function as transcriptional enhancers (Sawsan Khuri, personal communication); upon this basis, we interpret the low nucleosome occupancy of intragenic regions of the *SL6A3* gene as indicating that they might bear regulatory potential. Indeed, several intragenic NXScore peaks within the *SLC6A3*, including one in the 3′ UTR, have NXScore value about 600, suggesting that the open chromatin structure in these regions renders them susceptible to targeting by chromatin modifiers and possibly by other DNA interacting proteins.

The same computation assigns the NXScore to the promoter region that can serve as a proxy to the expression rate of a cognate gene. The NXScore value for the *SLC6A4* promoter (623) agrees well with the average score value for the promoters of the RefSeq human genes (about 600). Accordingly, the expression of the *SERT* is expected to be rather broad. The score predicted for the *SLC6A3* promoter (820) is significantly higher than the average, thus indicating that expression of the *SLC6A3* is not constitutive but contingent upon stimulation.

#### Histone modifications

DNA In cell nucleus is found in the form of a nucleoprotein, wherein histones represent the protein moiety. The N-terminal tail of the core histone proteins (H2A, H2B, H3 and H4) is a subject to various types of covalent modification, including methylation, acetylation, phosphorylation, sumoylation, and ubiquitination. Combinatorial arrangement of histone modifications determine the local packaging of the DNA, thereby influencing gene expression [127]. Acetylated histones mark actively transcribed genes. Enzymes with opposite functions, histone acetyltransferases (HATs) and histone deacetylases (HDACs), maintain the highly dynamics of histone acetylation/deacetylation status. Histone modifications are highly dynamic and reversible [128]; they are co-regulated with DNA methylation. For example, DNA methylation depends on the pre-established histone 3 methylation at lysine 9 [129] and aberrant DNA methylation occurs when the local pattern of histone modifications is distorted [130].

In the adult brain, changes in histone acetylation underlay synaptic plasticity [117]. Some drugs, including drugs of abuse, influence histone acetylation status; for example, administratered cocaine causes histone hyperacetylation [131] and, accordingly, clinical studies suggest that cocaine craving diminishes after treatment with valproate [132], [133], [134]. This evidence sheds some light on the pathogenesis of addiction: Since valproate has HDAC inhibiting activity, it is plausible that cocaine-mediated disregulation of gene that causes pathology is mitigated by the treatment. Both direct- and indirect- empirical evidence supports the involvement of epigenetic mechanisms in the *SLC6A3* regulation: Thus, it was established that valproic acid, acting via the Sp1 (see above), up-regulates the *SLC6A3* expression in a dose- and time-dependent manner [135]. In addition, researches using the ADHD animal model [136] reported that valproate attenuated hyperactive behaviors caused by the incomplete knockout of the *Slc6a3* gene. The fact that the *SLC6A3* is TATA-less (discussed above) must also be considered since TATA-less genes, generally, are biased towards regulation by HAT activity [125].

The collaborative efforts of many research groups have made experimental data on histone modification available via public domains. The high-throughput technologies, used to facilitate such analyses are very powerful yet suffer from limitations including the potential for inaccurately interpreting the biological significance of the data. Using cell lines as model systems implies that the culturing conditions affect their proliferation rate, transcriptional events and differentiation state. Apparently, the histone modification patterns that were detected in actively dividing cells could be partially related to malignant transformation hence, they can not be generalized to predict chromatin status i*n vivo*, especially when considering terminally differentiated post-mitotic cells such as neurons. However, this information may offer some basic guidelines. We retrieved a compendium of the available information via the UCSC Browser (Figure S 5). Histone modifications mapped to the *SLC6A4* locus corroborate well with the anticipated pattern (Figure S 5B): The TSS proximal region (+/− 2 kb) displays a characteristic combination of positive- and negative- histone marks. The *SLC6A3* promoter is strikingly different (Figure S 5A); here, the positive histone marks merely are present. It is possible that open chromatin conformation in 5′ to the *SLC6A4* TSS reflects its broader expression pattern whilst the lack of acetylated and tri-methylated histones in vicinity of the *SLC6A3* TSS suggests that the experimental cell lines do not express DAT.

#### Role of epigenetic mechanisms in transcription initiation

Histone modifications of the promoter region reflect the transcriptional state of genomic region, therefore the human core-promoter prediction software CoreBoost_HM integrates data on histone modification profiles with important sequence features. CoreBoost_HM is based on two classifiers, one for CpG related promoters and the other for non-CpG related promoters. We applied both classifiers to analyze the *SLC6A3* and *SLC6A4* so, to ascertain whether the epigenetic state of the regulatory region is critical for their transcription (Figure S 6A, B). As evident from the Figure, DNA methylation does not contributes equally to regulation of these genes. The *SLC6A3* promoter was detectable only when analyzed as CpG-dependent, while a set up for CpG island-independent promoter produced no results. In contrast, for the *SLC6A4*, both analyses detected a putative promoter in the region corresponding to the actual TSS.

## Discussion

The strikingly polymorphic DAT phenotype in humans and the biological mechanisms that connect individual genetic backgrounds with the phenotypes still are understood poorly. The functional demands on the *DAT1* regulation are seemingly contradictory since the vital necessity of assuring the robust functioning of the dopaminergic system coincides with the need for plasticity and rapid fluctuations in the transcription rate. To better understand the distinct sources and mechanisms that might facilitate a phenotypic diversity of this magnitude, we comprehensively interrogated the sequence of the *SLC6A3* gene applying computational methods.

Our analysis revealed **several** **unique features** **of the human** ***SLC6A3*** **gene sequence**, including (1) a very high frequency of SNPs (897, as in NCBI SNP) compared with the *SLC6A4* (441) and other brain-related genes, such as *BDNF* and *DRD4* (454 and 154 SNPs, respectively), that, depending on their positional effect, engender various biological consequences; (2) the abundance of VNTRs (more than 90 in the *SLC6A3* gene body alone), which is indicative of a tendency to open chromatin structure in the locus, and increased accessibility to chromatin modifiers; and (3) presence of intragenic CNVs. Most notable characteristic of the human *SLC6A3* is its high sensitivity of to **epigenetic regulation**: in contrast to the relative enrichment in GC nucleotides in the promoter-proximal region as occurs in most human genes, the entire *SLC6A3* locus has GC-bias sequence composition (0.55) and comprises multiple CpG sites comprising 27 bona fide CGIs (CpG islands).

Importantly, our analysis revealed that the distinctive sequence features of the *SLC6A3* gene recognized by an array of regulatory mechanisms are **evolutionally recent**. In fact, they are either uniquely human or shared between human and primates. We noted that the epigenetic sensitivity of the *DAT1* gene increased during evolution, and this process likely involves GC-bias gene conversion (GCBC) that is viewed as a major force driving genome evolution [137]. Indeed, whilst the mouse *Slc6A3* gene has GC content 0.46 and its promoter has no CpG island, promoter-overlapping CpG island is present in the rat's *Slc6a3* gene; furthering this tendency, the rhesus monkey's *SLC6A3* also has two intragenic CpG islands. During evolution, the increase in demands on the *DAT1* expression might well have driven changes in the gene sequence, expanding its sensitivity to the different regulatory mechanisms. In this view, the GC-density and the number of CGIs in the *DAT gene* might co-evolve with the growing role of epigenetic mechanisms in its control. In other words, the evolutional drift of the *SLC6A3* sequence towards accumulating GC-nucleotides might reflect its enhancing epigenetic potential, needed to accommodate regulatory demands, as dictated by the increasingly complex functions of the human brain.

The genomic regions experiencing GCBC are seen as recombination hotspots [137] and, as such, the inherent instability of the *SLC6A3* locus could explain its unique sequence features and regulatory attributes. These changes might be linked in time to the genome rearrangement event that resulted in the translocation of a syntenic block encompassing the *DAT* gene from the chromosome 13 (rodents) to the telomere-proximal region of the chromosome 5 (human and primates). In general, the propensity of genomic regions to instability is deleterious characteristic, but, theoretically, hypervariability arising from SNPs and tandem repeated sequences could be beneficial by allowing rapid adaptation [120], [138], [139]. Accordingly, the high variability of the *DAT* locus might support a broad basis for phenotypic diversity, and thus, be advantageous. Our detection of co-occurrence of the SNPs and the repeated sequences with the CGIs (116 SNPs and 2 tandem repeats) infers that the *DAT1* might still be evolving.

Our observation that there are several CNVs in the *SLC6A3* locus is not unique; they are detected in several human genes [140] that seemingly can asymptomatically tolerate this type of genetic variations. Here the human CNV genes stand apart; evidence from animal studies demonstrated that CNVs are depleted from laboratory mouse strains during selective breeding [141]. Consequently, it was postulated that that natural selection acts discriminately among human CNV genes and thus, at least for a subset of human genes, CNVs might confer adaptive benefits [140].

If propensity of the *DAT1* locus to instability is high and can give rise to frequent mutations, there must be other means to compensate for potentially sub-optimal gene function resulting from deleterious changes in its sequence. Considering the main concept of systems biology, that emphasizes the biological networks and hubs rather than a single specific process, we posit that the multi-modality of the *DAT* expressional control with multiple feed-back control loops ensures its functional robustness. Our analysis suggests that the **several arms of gene regulatory network** modulate the expression of the *SLC6A3*, whereby differential engagements and/or combinatorial interplay of different mechanisms of this network, DAT expression can be robust and reliable yet plastic and adjustable to accommodate environmental demands.

The novel regulatory paradigm for the *SLC6A3* gene that built upon our results might have direct practical implication. Acknowledging that DAT regulation is far more complex than that of average human gene implies that a comprehensive investigation is needed to reliably predict functional changes associated with the D*AT* polymorphism(s) and to infer possible phenotypic manifestations of sequence variation(s),. That is, we should consider broadening the scope of the future studies by interrogating the multiple features of the *SLC6A3* gene rather than focusing on any single polymorphism in its sequence. Also, the functional- and anatomical- diversity of the brain's dopamine system [61] must be accounted for. We elaborate this point taking the 3′-UTR VNTR polymorphism as an example. This polymorphism was investigated in relation to a variety of behavioral traits and diseases [142], [143], [144], and the findings of those research are conflicting. That is, the 10 repeats-allele (10/10 genotype) in some studies, i.e., [145] was associated with a lower translation rate and increased extracellular dopamine; while in the others, i.e., [146] with higher efficacy of the DAT translation *in vitro* and *in vivo* [147]. We suggest that considering a genomic position of the respective polymorphism might resolve this controversy. Indeed, the repeats number (the length of the polymorphic region can affect exclusively the longer mRNAs since the VNTR resides in the middle of 3′-UTR. Evidently, the shorter transcript isoforms (akin to S44625) are insensitive to this variation since here the transcription is terminated upstream of the VNTR. Considering the transcription bias towards preferential production of shorter isoforms in specific brain regions, we expect that a subset of brain functions executed through the specific brain regions is inherently independent of the polymorphism. Conversely, for the behavioral traits that rely on the transcription of long mRNA isoforms, innate differences in transcripts stability and conformational folding, subordinate to the genotype, will be manifest phenotypically.

We want to reiterate that the analyses and observations presented here are theoretical by nature and require experimental validation. However, we believe, that they help broaden our current understanding on the complex regulatory mechanisms imbedded in the human *DAT* gene sequence.

## Methods

The computational tools used to run the analyses and the databases used to retrieve the sequences and to visualize genomic features are listed in Table 1.

## Supporting Information

Figure S1 Copy number variations in the SLC6A3 locus. Information on validated structural variation maps of eight human genomes with resolution at the sequence level for selected regions is integrated in the UCSC Genome Browser. The CNVs in the SLC6A3 genetic locus are visualized in the Browser. The orange bar, indicating the longest deletion, encompasses two exons (indicated by arrows).
(0.10 MB EPS)

Figure S2 The SLC6A3 and the SLC6A4 regulatory regions. Red bar in this visual display represents cisRED annotation-based regulatory modules and predicted TFBSs are shown as brown blocks. In the DAT1 (top) red bar is long and predicted TFBSs are scattered over a long sequence stretch, whereas in the SERT (bottom), red bar is composed of discrete units and TFBSs are located within the TSS-proximal bar.
(0.09 MB EPS)

Figure S3 Clusters of short interspersed repeats in the DAT1 3′UTR. A - REPFIND program was used to identify clustered, exact repeats within the 5 kb region located on the 3′ - end of the SLC6A3 that includes 3′UTR. The program calculates a P-value indicating the probability of finding such a concentration of that particular repeat by chance for each repeat cluster found, and then selects the cluster with the most significant P-value. Top ten out of generated multiple repeated units with significant P-values multiple repeats found by the program are shown at the panel A. P-value of 8.18e-14 suggests that the closeness of these repeats is not random, and together they form a unit likely to have functional significance. B -CAG or CAG-containing motifs are the most abundant motif in 3′-UTRs of neuronal genes, therefore our identification of multiple CAG motif clusters in the 3′-UTR of the SLC6A3 posits that translation of its mRNAs is likely occur near the synaptic terminals. For this test, we used human 3′-UTR statistical background and P-value cutoff: 0.000001.
(0.19 MB EPS)

Figure S4 Secondary structure of the SLC6A3 transcript isoforms. A - The variation in the 3′UTR region of the SLC6A3 results in production of different length mRNAs. We used the GeneBee server to predict the secondary structure of RNAs transcribed from the sequences containing 10 versus 9 repeats which correspond to the most common allelic variations. As evident from the figure, there the presence of the 10th repeated increment profoundly changes the structure of the transcript. B - According to analysis of dyad symmetries (hairpins) predicted for the respective mRNA sequences (the SECentral Clone Manager Suite 7 program), the folding structure and hairpin formation generated by the 3′-UTR sequences containing 9 and 10 repeats are strikingly different. We found that the duplexes in 9 repeat's sequence have longer stems and higher energy than those from the 10 repeats (−14.4 kcals versus 7.4 kcals), suggesting that the 3′-UTR VNTR might influence the SLC6A3 gene expression by modulating the rate of mRNA degradation, which depends on conservation of the hairpin structures formed on mRNA 3′-UTR 's ends.
(0.10 MB EPS)

Figure S5 Experimental histone methylation data for the promoter region of the SLC6A3 and SLC6A4 genes. Compendium of these data is integrated in the UCSC genome browser, so, we retrieved the data on “active” histone modification mapped to the promoter regions of two genes. Histone profiling of the SLC6A3 (A) demonstrates lack of the acetylated histones and di-and tri-methylated H3K4 (green arrows), whereas the profile of the SLC6A4 regulatory region unequivocally reflects active status of the chromatin (red arrows) (B).
(0.17 MB EPS)

Figure S6 Prediction of the promoter region using CoreBoost_HM. We used two algorithms of the program to assess differences in the promoter prediction that depend on the epigenetic sensitivity. A - For the SLC6A3, density scores calculated for CpG-related promoters (upper panel) display the highest peak overlapping with the putative TSS (red arrow). Calculation under the assumption of CpG-independent promoter fails to produce a peak near the canonical TSS; and the position of the dominant peak is shifted downstream (green arrow). B - similar analyses carried out for the SLC6A4 gene showed that the position of the predicted promoter remains the same when CpG-association is considered (upper panel, red arrow) or discounted (lower panel, red arrow).
(0.13 MB EPS)

Table S1 Known SNPs in the SLC6A3 locus.
(0.20 MB PDF)

Table S2 Tandem repeats in the SLC6A3 locus with a period longer that 30 nt.
(0.03 MB PDF)

Table S3 Tandem repeats in the SLC6A4 locus.
(0.04 MB PDF)
