# Supplementary material for: Genomic Features of the Human Dopamine Transporter Gene and Its Potential Epigenetic States: Implications for Phenotypic Diversity
Source: PLoS One. 2010 Jun 10;5(6):e11067. doi: 10.1371/journal.pone.0011067 (PMC2883569; doi:10.1371/journal.pone.0011067)
Supplement: Table S1 — Known SNPs in the SLC6A3 locus. (0.20 MB PDF) [file pone.0011067.s007.pdf]

Table S 1 - SNPs in the SLC6A3 gene

SNP base (NCBI)	SNP rs ID	Alleles	Minor Allele	Minor-Allele Frequency (%)	Heterozygosity	Location	Conservation Score
1445965	rs7732456	C/ A	C	2	0.04	utr-3(dbSNP)	0
1446026	rs28363175	A/ G	A	1	0.01	utr-3(dbSNP)	0.005
1446038	rs28363174	A/ G	A	2	0.05	utr-3(dbSNP)	0.003
1446041	rs28363173	G/ C	G	1	0.01	utr-3(dbSNP)	0.003
1446063	rs1809939	G/ A	G	17	0.29	utr-3(dbSNP)	0.001
1446344	rs3797200	T/ C	T	16	0.28	utr-3(dbSNP)	0
1446454	rs16877691	G/ A	G	1	0.03	utr-3(dbSNP)	0.002
1446494	rs28363172	C/ T	C	1	0.01	utr-3(dbSNP)	0.002
1446511	rs28363171	A/ G	A	1	0.01	utr-3(dbSNP)	0
1446863	rs28363170	-/ GGGGGCCCTGCATGCGTCCTGG GGTAGTACACGCTCCAGT	-	13	0.23	utr-3(dbSNP)	0
1447234	rs28363169	T/ C	T	1	0.02	utr-3(dbSNP)	0
1447389	rs28363168	A/ C	A	3	0.07	utr-3(dbSNP)	0.004
1447522	rs27072	T/ C	T	20	0.31	utr-3(dbSNP)	0
1447600	rs11564774	G/ C	G	16	0.27	utr-3(dbSNP)	0
1447615	rs28363167	T/ C	T	1	0.01	utr-3(dbSNP)	0.003
1447814	rs28363166	T/ C	T	4	0.08	utr-3(dbSNP)	0
1447815	rs1042098	G/ A	G	28	0.4	utr-3(dbSNP)	0
1447938	rs28363165	-/ TGGCTAAGAGCAGCTGAAGG	-	1	0.01	intron(dbSNP)	0.002
1448077	rs40184	T/ C	T	42	0.49	intron(dbSNP)	0
1448113	rs28363164	T/ C	T	1	0.01	intron(dbSNP)	0
1448114	rs28363163	A/ G	A	1	0.01	intron(dbSNP)	0
1448115	rs28363162	T/ C	T	1	0.01	intron(dbSNP)	0
1448131	rs28363161	A/ G	A	2	0.04	intron(dbSNP)	0
1448391	rs28363160	A/ C	A	3	0.05	intron(dbSNP)	0
1449813	rs11564773	C/ T	C	11	0.19	intron(dbSNP)	0
1451007	rs11564772	T/ C	T	12	0.22	intron(dbSNP)	0.001
1451797	rs11564771	A/ G	A	13	0.23	intron(dbSNP)	0.11
1452431	rs11564769	C/ T	C	8	0.16	intron(dbSNP)	0
1452951	rs28363159	T/ C	T	1	0.03	intron(dbSNP)	0

1453192	rs28363158	C/ T	C	1	0.01	intron(dbSNP)	0
1453241	rs28363157	T/ A/ G	A	1	multiallelic	intron(dbSNP)	0
1453365	rs28363156	T/ C	T	1	0.02	intron(dbSNP)	0
1453368	rs10155607	G/ T	G	2	0.03	intron(dbSNP)	0
1453538	rs28363155	T/ G	T	1	0.02	intron(dbSNP)	0
1453596	rs28363154	G/ T/ C	T	1	multiallelic	intron(dbSNP)	0
1453685	rs28363153	T/ C	T	1	0.02	intron(dbSNP)	0
1453692	rs28363152	G/ T	G	1	0.02	intron(dbSNP)	0
1453750	rs28363151	A/ G	A	1	0.03	intron(dbSNP)	0
1453845	rs28363150	T/ C	T	1	0.01	intron(dbSNP)	0
1453931	rs28363149	TCTACACCAGCCCTG/ -	ACACCAGCC	12	0.22	intron(dbSNP)	0
1454140	rs28363148	A/ G	A	1	0.01	intron(dbSNP)	0
1454177	rs28363147	A/ C	A	1	0.01	intron(dbSNP)	0.005
1454314	rs28363146	T/ C	T	1	0.01	intron(dbSNP)	0.002
1454371	rs28363145	C/ T	C	1	0.01	intron(dbSNP)	0
1454380	rs28363144	T/ C	T	1	0.01	intron(dbSNP)	0
1454432	rs28363143	T/ C	T	1	0.01	intron(dbSNP)	0
1454483	rs28363142	A/ G	A	1	0.02	intron(dbSNP)	0
1454576	rs28363141	C/ T	C	3	0.05	intron(dbSNP)	0
1454580	rs11133767	T/ C	T	35	0.46	intron(dbSNP)	0
1454763	rs28363140	T/ C	T	1	0.01	intron(dbSNP)	0.001
1454828	rs28363139	-/ GCGCTGG	-	1	0.02	intron(dbSNP)	0
1455405	rs28363138	C/ G	C	2	0.05	intron(dbSNP)	0
1455548	rs28363137	T/ C	T	1	0.01	intron(dbSNP)	0.003
1455560	rs28363136	T/ G	T	1	0.01	intron(dbSNP)	0
1455630	rs28363135	A/ G	A	1	0.01	intron(dbSNP)	0
1455671	rs28363134	C/ G	C	1	0.01	intron(dbSNP)	0
1455757	rs28363133	C/ G	C	1	0.02	intron(dbSNP)	0.002
1455884	rs28363132	T/ C	T	1	0.01	intron(dbSNP)	0
1455971	rs28363131	C/ T	C	1	0.01	intron(dbSNP)	0
1456024	rs28363130	C/ T	C	1	0.01	intron(dbSNP)	0
1456073	rs6349	A/ G	A	5	0.09	coding-synonymous(dbSNP)	0.968
1456244	rs28363129	A/ G	A	1	0.02	intron(dbSNP)	0
1456615	rs28363128	G/ A	G	1	0.02	intron(dbSNP)	0
1457216	rs28363127	G/ T	G	1	0.01	intron(dbSNP)	0.227

1457256	rs28363126	A/ G	A	2	0.03	intron(dbSNP)	0
1457291	rs28363125	T/ C	T	1	0.02	intron(dbSNP)	0
1457466	rs11564768	G/ A	G	1	0.01	intron(dbSNP)	0
1457486	rs28363124	A/ G	A	2	0.03	intron(dbSNP)	0
1457548	rs6869645	T/ C	T	10	0.17	intron(dbSNP)	0
1457704	rs11564767	T/ A	T	19	0.3	intron(dbSNP)	0
1457726	rs11564766	T/ C	T	3	0.05	intron(dbSNP)	0
1457813	rs28363123	A/ G	A	1	0.01	intron(dbSNP)	0
1457922	rs11564765	C/ T	C	3	0.05	intron(dbSNP)	0.001
1458146	rs28363122	A/ G	A	1	0.01	intron(dbSNP)	0
1458473	rs28363121	T/ G	T	1	0.01	intron(dbSNP)	0
1458490	rs28363120	T/ C	T	14	0.24	intron(dbSNP)	0
1458694	rs28363119	C/ T	C	8	0.14	intron(dbSNP)	0
1458723	rs11133768	C/ T	C	11	0.19	intron(dbSNP)	0
1458725	rs28363118	G/ A	G	1	0.01	intron(dbSNP)	0
1458737	rs28363117	A/ G	A	3	0.06	intron(dbSNP)	0
1458781	rs28363116	T/ C	T	1	0.02	intron(dbSNP)	0
1458806	rs11564764	T/ C	T	3	0.07	intron(dbSNP)	0
1458882	rs28363115	T/ C	T	11	0.2	intron(dbSNP)	0
1458964	rs28363114	-/ CTTCGAC	-	3	0.05	intron(dbSNP)	0
1459036	rs6876225	A/ C	A	10	0.17	intron(dbSNP)	0
1459375	rs6880875	T/ C	T	8	0.16	coding-synonymous(dbSNP)	1
1460104	rs3776513	T/ G	T	12	0.22	intron(dbSNP)	0
1460116	rs3776512	A/ G	A	11	0.2	intron(dbSNP)	0
1460129	rs3776511	T/ C	T	12	0.21	intron(dbSNP)	0
1460151	rs28363113	G/ C	G	1	0.02	intron(dbSNP)	0.002
1460379	rs10072058	G/ A	G	10	0.18	intron(dbSNP)	0.001
1460419	rs28363112	T/ C	T	1	0.01	intron(dbSNP)	0.002
1460491	rs28363111	-/ A	-	16	0.27	intron(dbSNP)	0
1460634	rs28363110	A/ G	A	1	0.01	intron(dbSNP)	0
1461897	rs28363109	T/ C	T	1	0.02	intron(dbSNP)	0
1461979	rs11133770	C/ A	C	19	0.3	intron(dbSNP)	0
1462127	rs429699	T/ C	T	2	0.04	intron(dbSNP)	0
1462169	rs8179034	A/ G	A	1	0.01	coding-synonymous(dbSNP)	0.998

1462226	rs8179035	C/ G	C	1	0.01	coding-synonymous(dbSNP)	1
1462528	rs28363108	T/ C	T	1	0.01	intron(dbSNP)	0
1462693	rs28363107	A/ G	A	1	0.01	intron(dbSNP)	0
1462741	rs28363106	A/ G	A	2	0.05	intron(dbSNP)	0
1462836	rs2270912	A/ G	A	1	0.01	coding-synonymous(dbSNP)	0.996
1462985	rs8179029	T/ C	T	13	0.22	intron(dbSNP)	0
1463020	rs8179031	C/ T	C	2	0.04	intron(dbSNP)	0
1463060	rs28363105	T/ G	T	1	0.01	intron(dbSNP)	0
1463073	rs28363104	G/ A	G	2	0.04	intron(dbSNP)	0
1463147	rs28363103	T/ C	T	1	0.01	intron(dbSNP)	0
1463392	rs28363102	A/ C	A	1	0.01	intron(dbSNP)	0
1463472	rs2617577	C/ T	C	25	0.38	intron(dbSNP)	0
1463495	rs28363101	T/ C	T	3	0.05	intron(dbSNP)	0
1463571	rs28363100	T/ C	T	1	0.01	intron(dbSNP)	0
1463613	rs11564762	T/ G	T	14	0.24	intron(dbSNP)	0
1463948	rs28363099	C/ T	C	1	0.03	intron(dbSNP)	0
1463979	rs28363098	A/ G	A	2	0.04	intron(dbSNP)	0
1464025	rs28363097	A/ G	A	1	0.01	intron(dbSNP)	0
1464256	rs2550936	C/ A	C	37	0.46	intron(dbSNP)	0
1464412	rs6347	C/ T	C	38	0.47	coding-synonymous(dbSNP)	0.001
1465251	rs27047	C/ T	C	45	0.5	intron(dbSNP)	0
1465343	rs28363096	A/ G	A	1	0.01	intron(dbSNP)	0
1465361	rs28363095	A/ G	A	1	0.01	intron(dbSNP)	0
1465618	rs28363094	T/ G	T	1	0.01	intron(dbSNP)	0
1465645	rs27048	T/ C	T	35	0.45	intron(dbSNP)	0
1465664	rs28363093	T/ C	T	1	0.01	intron(dbSNP)	0
1465787	rs28363092	C/ G	C	1	0.02	intron(dbSNP)	0
1465793	rs28363091	G/ A	G	1	0.01	intron(dbSNP)	0.001
1465827	rs11564761	C/ -	C	1	0.01	intron(dbSNP)	0.001
1465848	rs28363090	A/ G	A	1	0.02	intron(dbSNP)	0
1465950	rs10052985	G/ A	G	7	0.13	intron(dbSNP)	0
1465971	rs28363089	A/ G	A	2	0.04	intron(dbSNP)	0
1465983	rs28363088	T/ C	T	1	0.01	intron(dbSNP)	0

1466130	rs28363087	T/ C	T	1	0.01	intron(dbSNP)	0
1466378	rs28363086	C/ T	C	1	0.02	intron(dbSNP)	0
1466567	rs28363085	A/ G	A	1	0.02	intron(dbSNP)	0
1466633	rs28363084	G/ A	G	1	0.01	intron(dbSNP)	0
1466756	rs28363083	G/ A	G	1	0.02	intron(dbSNP)	0
1466777	rs28363082	G/ A	G	1	0.02	intron(dbSNP)	0
1466787	rs28363081	G/ C	G	1	0.01	intron(dbSNP)	0
1466799	rs28363080	A/ G	A	2	0.03	intron(dbSNP)	0
1466903	rs28363079	A/ G	A	1	0.02	intron(dbSNP)	0
1466953	rs28363078	T/ C	T	2	0.04	intron(dbSNP)	0
1466991	rs28363077	T/ C	T	1	0.02	intron(dbSNP)	0
1467091	rs28363076	A/ G	A	2	0.04	intron(dbSNP)	0.001
1467160	rs28363075	T/ C	T	1	0.01	intron(dbSNP)	0
1467751	rs28363074	C/ G	C	1	0.02	intron(dbSNP)	0.001
1467756	rs28363073	T/ C	T	1	0.01	intron(dbSNP)	0
1467773	rs2963253	A/ G	A	4	0.08	intron(dbSNP)	0.002
1467876	rs28363072	A/ G	A	1	0.02	coding-synonymous(dbSNP)	1
1467951	rs28363071	A/ G	A	1	0.01	intron(dbSNP)	0
1468068	rs28363070	A/ G	A	1	0.01	intron(dbSNP)	0
1468190	rs28363069	A/ G	A	1	0.01	intron(dbSNP)	0
1468192	rs28363068	A/ G	A	1	0.01	intron(dbSNP)	0
1468257	rs28382258	A/ G	A	1	0.01	intron(dbSNP)	0
1468291	rs11564759	A/ G	A	25	0.37	intron(dbSNP)	0
1468340	rs28382257	A/ G	A	1	0.02	intron(dbSNP)	0
1468351	rs28382256	A/ G	A	1	0.01	intron(dbSNP)	0
1468361	rs28382255	T/ G	T	1	0.01	intron(dbSNP)	0
1468404	rs466630	G/ C	G	40	0.48	intron(dbSNP)	0
1468405	rs456645	G/ A	G	36	0.46	intron(dbSNP)	0
1468629	rs37022	T/ A	T	34	0.45	intron(dbSNP)	0
1468668	rs28382254	G/ T	G	1	0.02	intron(dbSNP)	0.151
1469044	rs28382253	A/ G	A	1	0.01	intron(dbSNP)	0
1469142	rs40358	A/ C	A	46	0.5	intron(dbSNP)	0
1469646	rs2042449	A/ G	A	18	0.3	intron(dbSNP)	0
1469894	rs28382252	G/ A	G	1	0.02	intron(dbSNP)	0
1469898	rs28382251	C/ G	C	1	0.02	intron(dbSNP)	0

1469984	rs28382250	A/ G	A	1	0.01	intron(dbSNP)	0
1470047	rs2963252	T/ C	T	3	0.06	intron(dbSNP)	0
1470212	rs13161905	T/ C	T	16	0.27	intron(dbSNP)	0
1470652	rs2975293	T/ C	T	4	0.07	intron(dbSNP)	0
1471374	rs37020	A/ C	A	37	0.47	intron(dbSNP)	0.001
1471558	rs10040882	T/ C	T	19	0.31	intron(dbSNP)	0
1472745	rs2927677	C/ T	C	1	0.03	intron(dbSNP)	0
1472932	rs2975292	G/ C	G	43	0.49	intron(dbSNP)	0.002
1473228	rs28382249	C/ T	C	1	0.01	intron(dbSNP)	0
1473268	rs2735917	A/ C	A	6	0.11	intron(dbSNP)	0
1473306	rs28382248	A/ G	A	24	0.37	intron(dbSNP)	0
1473346	rs28382247	T/ G	T	21	0.33	intron(dbSNP)	0.001
1473382	rs28382246	A/ T	A	24	0.36	intron(dbSNP)	0
1473476	rs28382245	T/ C	T	25	0.37	intron(dbSNP)	0
1473588	rs11564758	G/ C	G	18	0.29	intron(dbSNP)	0.004
1473801	rs6348	A/ G	A	10	0.18	coding-synonymous(dbSNP)	1
1474184	rs2975291	T/ G	T	1	0.02	intron(dbSNP)	0.009
1474197	rs28382244	-/ G	-	2	0.03	intron(dbSNP)	0
1474228	rs28382243	T/ C	T	1	0.01	intron(dbSNP)	0.004
1474543	rs11133778	G/ C	G	24	0.36	intron(dbSNP)	0.001
1474562	rs28382242	C/ T	C	1	0.02	intron(dbSNP)	0.021
1474709	rs28382241	C/ G	C	1	0.01	intron(dbSNP)	0
1474732	rs2975289	T/ C	T	1	0.01	intron(dbSNP)	0
1474907	rs28382240	T/ C	T	1	0.01	intron(dbSNP)	0.001
1476905	rs464049	A/ G	A	38	0.47	intron(dbSNP)	0
1478159	rs250686	T/ C	T	42	0.49	intron(dbSNP)	0
1480803	rs250682	C/ G	C	43	0.49	intron(dbSNP)	0
1481011	rs250681	C/ T	C	37	0.47	intron(dbSNP)	0.001
1481111	rs10052016	G/ A	G	18	0.29	intron(dbSNP)	0.002
1481135	rs10053602	C/ T	C	18	0.29	intron(dbSNP)	0.003
1481514	rs393795	T/ G	T	37	0.47	intron(dbSNP)	0
1482969	rs460700	C/ T	C	34	0.45	intron(dbSNP)	0
1483093	rs458334	A/ G	A	38	0.47	intron(dbSNP)	0
1483244	rs464061	A/ G	A	38	0.47	intron(dbSNP)	0.001
1483515	rs456082	G/ A	G	38	0.47	intron(dbSNP)	0

1483616	rs11737901	T/ C	T	16	0.27	intron(dbSNP)	0.001
1483662	rs28382239	C/ T	C	1	0.01	intron(dbSNP)	0
1483775	rs410209	G/ A	G	33	0.44	intron(dbSNP)	0
1483834	rs409588	T/ G	T	38	0.47	intron(dbSNP)	0
1483912	rs3834250	-/ TCACCTCCCGCCTGGCTGTTCCT	-	3	0.06	intron(dbSNP)	0.003
1483933	rs458860	A/ T	A	38	0.47	intron(dbSNP)	0.005
1484123	rs464528	T/ C	T	40	0.48	intron(dbSNP)	0.002
1484164	rs463379	C/ G	C	42	0.49	intron(dbSNP)	0
1484186	rs3056480	-/ CTC	-	8	0.15	intron(dbSNP)	0
1484214	rs461753	T/ C	T	37	0.47	intron(dbSNP)	0
1484302	rs458632	G/ A	G	31	0.43	intron(dbSNP)	0
1484306	rs460934	G/ A	G	1	0.02	intron(dbSNP)	0
1484554	rs462053	C/ T	C	34	0.45	intron(dbSNP)	0
1484622	rs457894	C/ T	C	34	0.45	intron(dbSNP)	0
1484853	rs460007	A/ G	A	31	0.43	intron(dbSNP)	0
1484992	rs461677	C/ A	C	32	0.43	intron(dbSNP)	0.005
1485042	rs459141	T/ C	T	32	0.43	intron(dbSNP)	0
1485164	rs28382237	A/ G	A	1	0.02	intron(dbSNP)	0
1485202	rs456774	C/ G	C	38	0.47	intron(dbSNP)	0
1485490	rs28382236	T/ C	T	1	0.01	intron(dbSNP)	0.001
1485552	rs2975284	A/ G	A	4	0.08	intron(dbSNP)	0
1485566	rs16878300	A/ G	A	2	0.03	intron(dbSNP)	0
1485825	rs460000	T/ G	T	38	0.47	intron(dbSNP)	NA
1485876	rs465130	A/ G	A	33	0.44	intron(dbSNP)	0.002
1485881	rs465989	T/ C	T	31	0.43	intron(dbSNP)	0
1485922	rs28382235	C/ A	C	1	0.01	intron(dbSNP)	0
1485966	rs3842031	G/ -	G	33	0.44	intron(dbSNP)	0.001
1486073	rs28382234	C/ T	C	2	0.04	intron(dbSNP)	0.004
1486401	rs4975646	A/ G	A	13	0.22	intron(dbSNP)	0
1487075	rs410481	T/ C	T	1	0.02	intron(dbSNP)	0.002
1489408	rs420422	T/ C	T	42	0.49	intron(dbSNP)	0.003
1490726	rs2963242	T/ C	T	6	0.12	intron(dbSNP)	0
1491105	rs1048955	C/ T	C	3	0.06	intron(dbSNP)	0
1491174	rs1048953	G/ A	G	13	0.22	intron(dbSNP)	0
1491354	rs403636	A/ C	A	21	0.33	intron(dbSNP)	0
1493280	rs246997	T/ G	T	19	0.31	intron(dbSNP)	0.022

1493504	rs28382233	A/ G	A	1	0.01	intron(dbSNP)	0.029
1493506	rs2963239	G/ A	G	4	0.07	intron(dbSNP)	0.029
1493596	rs28382232	T/ G	T	1	0.01	intron(dbSNP)	0
1493599	rs29001606	-/ AATAGATGATTGACAGAT	-	1	0.01	intron(dbSNP)	0
1493620	rs28742609	-/ AATA	-	1	0.01	intron(dbSNP)	0.001
1493681	rs2927676	G/ C	G	21	0.33	intron(dbSNP)	0.001
1493728	rs2927675	A/ G	A	3	0.06	intron(dbSNP)	0.01
1493934	rs28742608	G/ T	G	5	0.1	intron(dbSNP)	0
1494162	rs28382231	G/ A	G	1	0.01	intron(dbSNP)	0.001
1494261	rs28382230	A/ C	A	1	0.02	intron(dbSNP)	0
1494281	rs28382229	G/ A	G	1	0.01	intron(dbSNP)	0
1494306	rs28382228	T/ C	T	3	0.05	intron(dbSNP)	0.012
1494436	rs2911492	A/ G	A	2	0.05	intron(dbSNP)	0
1494711	rs28382227	T/ C	T	1	0.01	intron(dbSNP)	0
1494951	rs28382226	T/ C	T	1	0.02	intron(dbSNP)	0
1494997	rs28382225	G/ C	G	1	0.01	intron(dbSNP)	0
1495521	rs2617605	C/ T	C	27	0.39	intron(dbSNP)	0
1495552	rs28382224	T/ C	T	1	0.01	intron(dbSNP)	0
1495654	rs28382223	C/ G	C	1	0.01	intron(dbSNP)	0.004
1495676	rs28382222	A/ G	A	2	0.04	intron(dbSNP)	0
1495732	rs2981359	C/ G	C	49	0.5	intron(dbSNP)	0.001
1495757	rs28382221	T/ C	T	7	0.14	intron(dbSNP)	0
1495842	rs13189021	T/ C	T	8	0.15	intron(dbSNP)	0
1495974	rs2254408	A/ C	A	39	0.48	intron(dbSNP)	0.001
1495979	rs2270914	T/ C	T	19	0.31	intron(dbSNP)	0
1495987	rs2270913	A/ G	A	6	0.11	intron(dbSNP)	0
1496007	rs8179023	C/ G	C	16	0.26	intron(dbSNP)	0
1496151	rs6351	A/ G	A	1	0.01	coding-synonymous(dbSNP)	0
1496163	rs6346	A/ C	A	4	0.08	coding-synonymous(dbSNP)	0
1496199	rs6350	A/ G	A	6	0.12	coding-synonymous(dbSNP)	0.99
1496498	rs2455391	A/ G	A	22	0.34	intron(dbSNP)	0

1496603	rs2975223	C/ T	C	45	0.49	intron(dbSNP)	0
1496604	rs2937640	T/ C	T	45	0.5	intron(dbSNP)	0
1496607	rs28382220	T/ C	T	1	0.01	intron(dbSNP)	0
1496728	rs2937639	C/ T	C	44	0.49	intron(dbSNP)	0
1496762	rs11564757	T/ C	T	6	0.11	intron(dbSNP)	0
1496763	rs28382219	C/ A	C	1	0.01	intron(dbSNP)	0
1496990	rs1316830	T/ C	T	39	0.47	intron(dbSNP)	0
1496998	rs28382218	A/ G	A	1	0.02	intron(dbSNP)	0
1497218	rs28382217	A/ G	A	1	0.01	intron(dbSNP)	0
1497261	rs28382216	A/ G	A	1	0.01	intron(dbSNP)	0
1497427	rs2963238	G/ T	G	47	0.5	intron(dbSNP)	0
1497581	rs28382215	G/ A	G	1	0.01	intron(dbSNP)	0.002
1497817	rs2911493	T/ C	T	2	0.05	intron(dbSNP)	0.001
1497970	rs28382214	T/ C	T	1	0.01	intron(dbSNP)	0
1497985	rs11564752	A/ C	A	5	0.09	intron(dbSNP)	0
1498379	rs2735854	T/ C	T	2	0.04	intron(dbSNP)	0
