# Supplementary material for: Genomic Features of the Human Dopamine Transporter Gene and Its Potential Epigenetic States: Implications for Phenotypic Diversity
Source: PLoS One. 2010 Jun 10;5(6):e11067. doi: 10.1371/journal.pone.0011067 (PMC2883569; doi:10.1371/journal.pone.0011067)
Supplement: Table S2 — Tandem repeats in the SLC6A3 locus with a period longer that 30 nt. (0.03 MB PDF) [file pone.0011067.s008.pdf]

*Table S 2 - Tandem repeats in the SLC6A3 (period more than 30 bp)*

| ***VARscore*** | ***Start*** | ***End*** | ***Period*** | ***Exponent*** | ***Consensus length*** | ***% match*** | ***% indels*** | ***TRFscore*** |
|---|---|---|---|---|---|---|---|---|
| -0.028704114 | 951 | 1036 | 44 | 2 | 44 | 90 | 0 | 144 |
| -1.034923948 | 966 | 1062 | 36 | 2.8 | 34 | 73 | 11 | 105 |
| -0.474704592 | 7919 | 8066 | 32 | 4.7 | 31 | 75 | 12 | 143 |
| ***2.821961676*** | 9296 | 9744 | 66 | 6.8 | 66 | 95 | 1 | 841 |
| -1.833366673 | 9296 | 9744 | 195 | 2.3 | 197 | 94 | 2 | 832 |
| ***1.223656129*** | 10224 | 10451 | 73 | 3.1 | 73 | 89 | 0 | 360 |
| -1.050985237 | 11176 | 11233 | 30 | 1.9 | 30 | 86 | 6 | 90 |
| ***1.034533915*** | 13637 | 14068 | 29 | 14.9 | 28 | 75 | 10 | 365 |
| -0.114151967 | 13632 | 14068 | 87 | 5 | 85 | 76 | 6 | 460 |
| ***1.213043456*** | 13632 | 14068 | 58 | 7.5 | 58 | 78 | 3 | 469 |
| 4.127747106 | 22042 | 23072 | 38 | 27.7 | 38 | 91 | 2 | 1612 |
| ***2.432854578*** | 22043 | 23072 | 74 | 13.8 | 75 | 91 | 2 | 1595 |
| -1.537137083 | 22042 | 23072 | 112 | 9.2 | 112 | 91 | 2 | 1634 |
| -1.132063425 | 23665 | 23760 | 29 | 3.3 | 29 | 68 | 7 | 98 |
| -0.06599552 | 26056 | 26867 | 82 | 10 | 80 | 66 | 19 | 338 |
| 0.839078649 | 28027 | 28310 | 54 | 5.2 | 54 | 81 | 3 | 355 |
| -1.424298858 | 28014 | 28310 | 109 | 2.7 | 109 | 83 | 1 | 437 |
| ***2.393938188*** | 28402 | 28929 | 55 | 9.6 | 55 | 83 | 4 | 688 |
| 0.026211934 | 30906 | 31044 | 38 | 3.6 | 38 | 91 | 0 | 222 |
| -0.139101424 | 30906 | 31068 | 38 | 4.2 | 38 | 85 | 2 | 214 |
| 0.535117174 | 33502 | 33683 | 30 | 6.1 | 30 | 98 | 0 | 350 |
| -0.748525789 | 41933 | 42048 | 40 | 2.7 | 44 | 80 | 13 | 163 |
| 0.377474801 | 41872 | 42133 | 70 | 3.8 | 69 | 75 | 8 | 256 |
| -0.116118952 | 41946 | 42138 | 40 | 5.1 | 39 | 79 | 11 | 198 |
| ***2.310554075*** | 51439 | 51842 | 40 | 10 | 40 | 92 | 3 | 679 |
| -1.833320753 | 51439 | 51842 | 165 | 2.5 | 160 | 93 | 3 | 700 |
| -0.404375771 | 52326 | 52528 | 38 | 5 | 39 | 75 | 16 | 246 |
| -0.2794409 | 52313 | 52522 | 44 | 5 | 43 | 75 | 16 | 262 |
| 0.553927933 | 52313 | 52528 | 82 | 2.6 | 82 | 94 | 1 | 378 |
