# Supplementary material for: Genomic Features of the Human Dopamine Transporter Gene and Its Potential Epigenetic States: Implications for Phenotypic Diversity
Source: PLoS One. 2010 Jun 10;5(6):e11067. doi: 10.1371/journal.pone.0011067 (PMC2883569; doi:10.1371/journal.pone.0011067)
Supplement: Table S3 — Tandem repeats in the SLC6A4 locus. (0.04 MB PDF) [file pone.0011067.s009.pdf]

Table S3- tandem repeats in the SLC6A4

VARscore	*start*	*end*	*period*	*exponent*	*consensus*	*length*	*%match*	*%indels*	*TRFscore*
-1.018909256	5083	5114	17	1.9	17	87	6	52	31
-0.92775213	5089	5137	23	2.1	23	78	14	60	32
-0.87664277	5111	5158	14	3.5	14	71	18	46	39
-1.03244375	5112	5175	28	2.2	31	75	16	80	39
-0.561456817	5196	5298	***36***	2.8	36	88	5	152	45
-1.917650942	5119	5421	2	150	2	59	10	95	39
-0.191163122	5940	5967	5	5.6	5	86	0	42	14
1.210264534	6524	6674	3	50	3	87	1	176	0
1.049641397	9203	9234	2	16.5	2	87	6	52	56
-0.839116377	9795	9824	10	2.9	11	85	14	50	0
-0.832511234	9795	9824	11	2.8	12	85	14	45	0
-1.034821736	9965	9998	14	2.7	13	73	17	44	17
-1.083216714	14618	14644	14	1.9	14	100	0	54	22
0.934215772	15108	15313	17	12.4	16	87	7	314	7
0.357513953	15108	15313	***33***	6.2	32	90	5	314	7
1.357956456	15108	15313	***50***	4.1	50	90	3	332	7
-1.136841735	18578	18597	10	2	10	100	0	40	30
0.970082361	19820	19863	4	11.5	4	95	4	78	79
-1.160640731	20011	20035	13	1.9	13	91	0	43	84
0.827051746	20381	20446	5	13.2	5	77	16	82	0
1.042712779	21610	21765	4	38.8	4	81	11	201	68
-0.744394463	22046	22081	9	3.4	10	82	17	46	86
-0.80962617	22046	22081	14	2.6	14	82	4	46	86
0.382017668	22936	22963	4	7	4	100	0	56	75
-1.072768062	27009	27035	11	2.3	12	93	6	49	44
-0.743941181	27990	28026	10	3.8	10	75	7	48	75
-0.19317793	28097	28120	5	4.8	5	100	0	48	0
-0.553236874	28526	28590	20	3.2	20	84	0	102	20
-1.133536242	28738	28762	13	2	13	92	7	45	8
-0.588711622	30427	30463	11	3.5	11	85	14	57	0

-0.909971739	30425	30463	18	2.1	19	85	4	59	0
-1.045964301	30425	30463	20	1.9	20	89	0	64	0
0.501021163	30751	30782	4	8.2	4	93	6	59	78
-0.568394706	30751	30790	12	3.5	11	89	6	59	75
-0.08228808	31343	31363	4	5.2	4	100	0	42	0
-0.988723652	31485	31518	16	2.3	16	76	14	46	47
-0.804275721	36940	37005	19	3.2	19	73	22	83	25
